# Phosphate-mediated coanchoring of RBD immunogens and molecular adjuvants to alum potentiates humoral immunity against SARS-CoV-2

**DOI:** 10.1126/sciadv.abj6538

**Published:** 2021-12-08

**Authors:** Kristen A. Rodrigues, Sergio A. Rodriguez-Aponte, Neil C. Dalvie, Jeong Hyun Lee, Wuhbet Abraham, Diane G. Carnathan, Luis E. Jimenez, Julia T. Ngo, Jason Y. H. Chang, Zeli Zhang, Jingyou Yu, Aiquan Chang, Catherine Nakao, Benjamin Goodwin, Christopher A. Naranjo, Libin Zhang, Murillo Silva, Dan H. Barouch, Guido Silvestri, Shane Crotty, J. Christopher Love, Darrell J. Irvine

**Affiliations:** 1 Koch Institute for Integrative Cancer Research, Massachusetts Institute of Technology, Cambridge, MA 02139, USA.; 2 Harvard-MIT Health Sciences and Technology Program, Institute for Medical Engineering and Science, Massachusetts Institute of Technology, Cambridge, MA 02139, USA.; 3 Ragon Institute of Massachusetts General Hospital, Massachusetts Institute of Technology and Harvard University, Cambridge, MA 02139, USA.; 4 Consortium for HIV/AIDS Vaccine Development, The Scripps Research Institute, La Jolla, CA 92037, USA.; 5 Department of Biological Engineering, Massachusetts Institute of Technology, Cambridge, MA 02139, USA.; 6 Department of Chemical Engineering, Massachusetts Institute of Technology, Cambridge, MA 02139, USA.; 7 Center for Infectious Disease and Vaccine Research, La Jolla Institute for Immunology, La Jolla, CA 92037, USA.; 8 Yerkes National Primate Research Center, Emory University, Atlanta, GA 30322, USA.; 9 Emory Vaccine Center, Emory University School of Medicine, Atlanta, GA 30322, USA.; 10 Center for Virology and Vaccine Research, Beth Israel Deaconess Medical Center, Harvard Medical School, Boston, MA 02215, USA.; 11 Harvard Medical School, Boston, MA 02215, USA.; 12 Department of Materials Science and Engineering, Massachusetts Institute of Technology, Cambridge, MA 02139, USA.; 13 Howard Hughes Medical Institute, Chevy Chase, MD 20815, USA.

## Abstract

There is a need for additional rapidly scalable, low-cost vaccines against severe acute respiratory syndrome coronavirus 2 (SARS-CoV-2) to achieve global vaccination. Aluminum hydroxide (alum) adjuvant is the most widely available vaccine adjuvant but elicits modest humoral responses. We hypothesized that phosphate-mediated coanchoring of the receptor binding domain (RBD) of SARS-CoV-2 together with molecular adjuvants on alum particles could potentiate humoral immunity by promoting extended vaccine kinetics and codelivery of vaccine components to lymph nodes. Modification of RBD immunogens with phosphoserine (pSer) peptides enabled efficient alum binding and slowed antigen clearance, leading to notable increases in germinal center responses and neutralizing antibody titers in mice. Adding phosphate-containing CpG or saponin adjuvants to pSer-RBD:alum immunizations synergistically enhanced vaccine immunogenicity in mice and rhesus macaques, inducing neutralizing responses against SARS-CoV-2 variants. Thus, phosphate-mediated coanchoring of RBD and molecular adjuvants to alum is an effective strategy to enhance the efficacy of SARS-CoV-2 subunit vaccines.

## Teaser

Coanchoring a SARS-CoV-2 immunogen and molecular adjuvants to alum extends vaccine kinetics and amplifies humoral immunity.

## INTRODUCTION

The coronavirus disease 2019 (COVID-19) pandemic has been caused by the emergence of a novel betacoronavirus, severe acute respiratory syndrome coronavirus 2 (SARS-CoV-2), leading to more than 200 million confirmed cases and 4 million deaths worldwide. Viral entry into cells is mediated by the receptor binding domain (RBD) of the SARS-CoV-2 Spike (S) protein, which binds the human angiotensin-converting enzyme 2 (hACE2) receptor (*1*, *2*). Neutralizing antibody responses against the S protein or the RBD have been shown to protect against SARS-CoV-2 infection in animal models (*3*–*5*) and are believed to be a correlate of protection against SARS-CoV-2 (*6*–*9*), making the RBD an attractive vaccine target for neutralizing responses (*9*–*12*).

Although safe and effective vaccines are already being deployed in many developed nations, there remains a substantial need for strategies to facilitate global SARS-CoV-2 vaccine coverage. To this end, subunit vaccines are attractive for their ability to be produced at low cost, at scale and without the need for ultracold storage temperatures, but the global supply of adjuvants for accessible vaccines is unclear. The most common clinical vaccine adjuvant, alum, is well suited to global vaccination campaigns because of its manufacturability and low cost, but alum has exhibited relatively poor immunogenicity with SARS-CoV-2 subunit vaccines to date (*10*, *11*). Of equal importance to these practical issues is the ability of vaccines to promote neutralizing responses to SARS-CoV-2 variants that are now circulating globally (*13*–*16*). A number of preclinical studies have demonstrated that vaccines eliciting higher levels of neutralizing responses against the original Wuhan-Hu-1 virus tend to also elicit high neutralizing titers against these viral variants (*17*–*21*). Hence, approaches to enhancing the immunogenicity of alum-adjuvanted subunit vaccines for SARS-CoV-2 may be important in the effort to achieve global vaccination coverage.

Inspired by earlier fundamental work examining the interactions of phosphorylated proteins with aluminum hydroxide (*22*–*25*), we recently described an approach to augmenting alum:protein subunit vaccines by site-specific introduction of phosphoserine (pSer) peptide tags onto protein immunogens (*26*). pSer tagging allows immunogens to bind to the surface of aluminum hydroxide via a ligand exchange reaction, providing tight binding that can be tuned by the valency of the pSer peptide tag sequence. Stable anchoring to alum was shown to prolong antigen delivery to lymph nodes via slow trafficking of alum particles, coincident with direct B cell triggering by antigen multivalently displayed on alum. These changes in the physical chemistry of vaccine delivery enhanced germinal center (GC) responses, serum antibody titers, and neutralizing antibody titers against HIV envelope (Env) immunogens (*26*).

Despite these promising data, alum remains an adjuvant that does not stimulate many of the innate immune recognition pathways that might be exploited to drive robust immune responses. We hypothesized that phosphate-mediated binding could be used to coanchor SARS-CoV-2 antigens and complementary molecular adjuvants to alum particles to synergistically drive humoral immunity. To test this idea, we evaluated the potential of pSer tagging to enhance the immunogenicity of alum:RBD subunit vaccines in mice and rhesus macaque (RM) nonhuman primates (NHPs). We assessed alum binding, antigen structural stability, and in vivo humoral immune responses for pSer-modified RBD proteins. Immunization with pSer-labeled RBD antigens was found to greatly enhance the immunogenicity of this antigen in combination with alum. To further amplify these responses, we combined pSer-tagged RBD with the Toll-like receptor 9 (TLR9) ligand CpG or a saponin/phospholipid nanoparticle adjuvant [saponin-MPLA (monophosphoryl lipid A) nanoparticle (SMNP)] that intrinsically contains phosphate residues (*27*), for coadsorption to alum. We found that the persistence of these adjuvants in vivo could be significantly increased by complexing with pSer-RBD and alum, correlating with synergistic enhancements in vaccine immunogenicity. In a pilot study in RMs, pSer-RBD:alum:SMNP elicited robust neutralizing antibody responses that cross-reacted with SARS-CoV-2 variants of concern. These findings suggest that pSer modification may be a promising way to enhance the efficacy of SARS-CoV-2 subunit vaccines and that combining alum with molecular adjuvants capable of undergoing ligand exchange–mediated binding can further substantially potentiate humoral immunity.

## RESULTS

### pSer peptide modification facilitates stable binding of SARS-CoV-2 RBD to alum

**Fig. 1.**
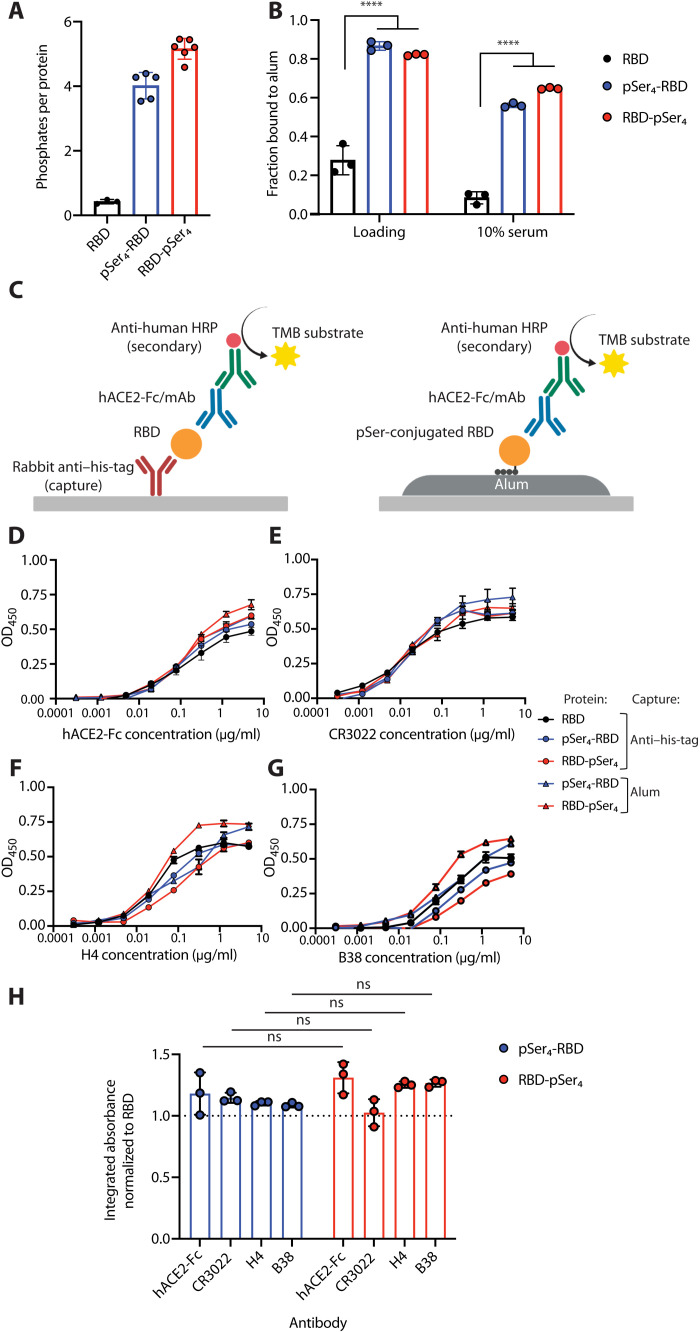
pSer modification of SARS-CoV-2 RBD immunogens facilitates binding to alum with retention of key structural epitopes. (**A**) RBD antigens with pSer peptides conjugated at the N terminus (pSer_4_-RBD) or C terminus (RBD-pSer_4_) were assayed for phosphates per protein by a malachite green assay. (**B**) pSer-conjugated or unmodified RBD was mixed with alum, and the fraction of protein bound to alum was assessed before (loading) and after incubation for 24 hours in 10% mouse serum at 37°C. Statistical significance was determined by one-way analysis of variance (ANOVA) followed by Tukey’s post hoc test. (**C**) Schematic of modified sandwich ELISA to analyze the antigenicity profile of free RBD (left) or RBD bound to alum-coated plates (right). TMB, 3,3′,5,5′- tetramethylbenzidine. (**D** to **F**) Shown are binding profiles of hACE2-Fc (D), CR3022 (E), H4 (F), and B38 (**G**) to RBDs captured on anti–his-tag or alum-coated plates (*n* = 3 replicates) and the area under individual binding curves normalized to unmodified RBD (**H**). Dotted line indicates signal equivalent to unmodified RBD. OD_450_, optical density at 450 nm. Statistical significance was determined by Mann-Whitney test. Values plotted are means ± SD. Not significant (ns), *P* > 0.05; ****P < 0.0001.

We first tested whether coupling a pSer peptide tag to Wuhan-Hu-1 SARS-CoV-2 RBD could engender stable binding to alum without disrupting key epitopes on the antigen. RBD (amino acids 332 to 532 of SARS-CoV-2 S protein; table S1) modified with a his-tag for purification and containing an N- or C-terminal free cysteine was expressed in yeast and then conjugated with a peptide tag containing a maleimide group linked to a six-unit poly(ethylene glycol) spacer, followed by four pSer residues (fig. S1). Measurement of the mean number of phosphates per protein using a malachite green assay revealed that the N terminus–coupled pSer_4_-RBD and C terminus–modified RBD-pSer_4_ gained the expected ~4 phosphates per protein (Fig. 1A). Incubation of unmodified RBD with alum in tris-buffered saline (TBS) led to adsorption of only ~25% of the antigen (Fig. 1B, “loading”), most of which desorbed following a 24-hour incubation of the alum in phosphate buffer containing 10% serum (Fig. 1B, “10% serum”). By contrast, both pSer-RBDs exhibited high levels of alum binding in buffer, with most remaining bound after serum/phosphate exposure (Fig. 1B). To confirm that the alum-bound RBD conjugates were structurally intact relative to unmodified protein, we used a modified sandwich enzyme-linked immunosorbent assay (ELISA) approach to probe the antigenicity of the constructs. Unmodified RBD was captured on plates coated with a his-tag–specific antibody, while pSer-conjugated RBD was captured on alum-coated plates (Fig. 1C). The immobilized RBD was then probed for binding to serial dilutions of recombinant hACE2 protein, the target receptor recognized by RBD, or monoclonal antibodies CR3022 [which recognizes a highly conserved epitope distal from the receptor binding site (*28*)], H4, or B38. As shown in Fig. 1 (D to H), the pSer-modified RBDs had antigenicity profiles indistinguishable from unmodified RBD, and the proteins captured on alum retained recognition of both probes. Thus, pSer modification allowed substantially enhanced RBD binding to alum without disrupting its structure.

### N-terminal pSer modification enhances the immunogenicity of RBD antigens

**Fig. 2.**
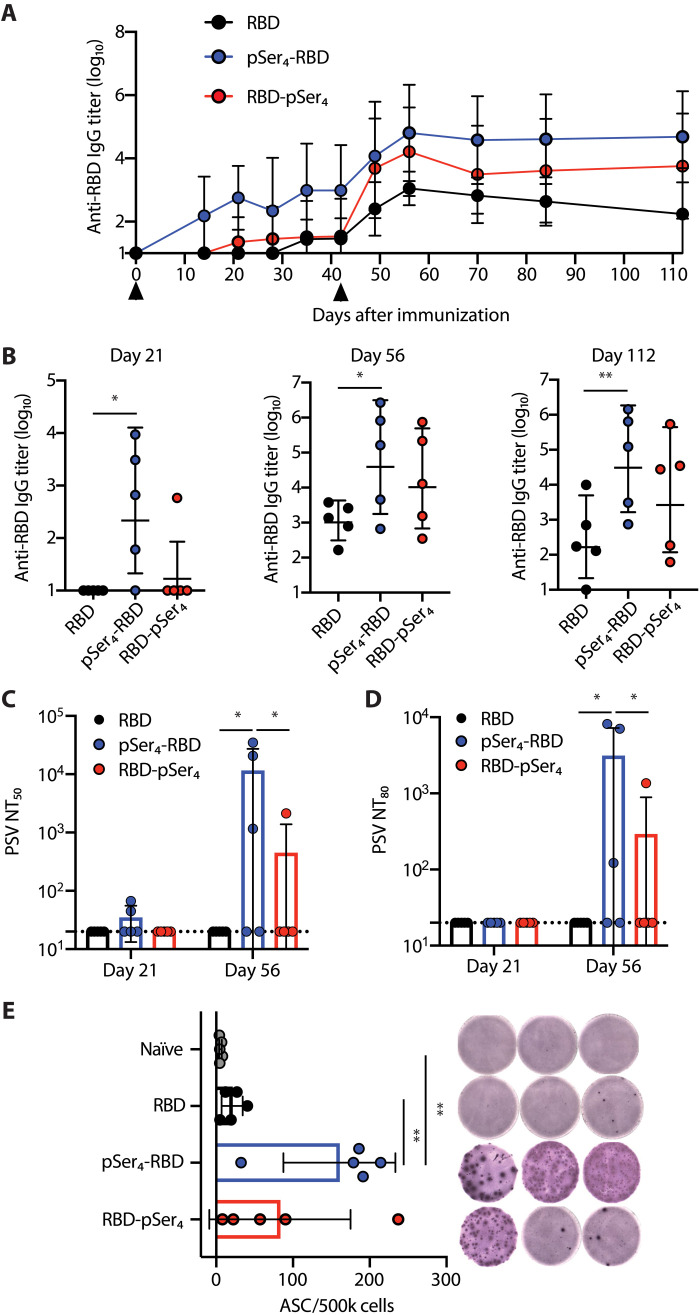
pSer modification enhances the immunogenicity of alum-adsorbed RBD in mice. BALB/c mice (*n* = 5 animals per group) were immunized with 10 μg of unmodified or N- or C-terminal pSer_4_-conjugated RBD in 50 μg of Alhydrogel and boosted at 6 weeks. (**A**) Serum IgG responses were assessed longitudinally by ELISA. Arrowheads indicate immunization time points. Values plotted are geometric means ± geometric SD. (**B**) Individual mouse IgG responses from selected time points. Values plotted are geometric means ± geometric SD. Statistical significance was determined by two-way ANOVA followed by Tukey’s post hoc test. SARS-CoV-2 PSV NT_50_ (**C**) and NT_80_ (**D**) were assessed for serum collected at days 21 and 56. The dotted line indicates the limit of detection (LOD). Values plotted are means ± SD. Statistical significance was determined by two-way ANOVA followed by Tukey’s post hoc test. (**E**) RBD-specific antibody-secreting cells (ASCs) in the bone marrow were assessed by enzyme-linked immune absorbent spot (ELISpot) at day 112. Representative ELISpot plate images are shown. Values plotted are means ± SD. Statistical significance was determined by one-way ANOVA followed by Tukey’s post hoc test. ns, *P* > 0.05; *P < 0.05; **P < 0.01.

We immunized BALB/c mice with pSer_4_-RBD, RBD-pSer_4_, or unmodified RBD combined with alum and boosted at 6 weeks. Consistent with prior reports (*10*, *11*), RBD:alum immunization elicited weak immunoglobulin G (IgG) responses, with none of the animals seroconverting by 3 weeks after prime at this dose; after boost, weak IgG titers were detected that steadily declined over time (Fig. 2A). Both pSer-modified immunogens exhibited stronger serum responses, and the N-terminally modified RBD was particularly effective, with titers 57-fold greater than the control group at the peak of response 2 weeks after boost (Fig. 2, A and B). Furthermore, traditional alum:RBD immunization elicited no detectable neutralizing responses even after boosting, while three of five animals receiving pSer_4_-RBD:alum vaccination had a pseudo-virus (PSV) neutralizing titer ID_50_ (50% inhibitory dose) (NT_50_) of >10^3^ after boost (Fig. 2, C and D). pSer_4_-RBD also significantly augmented the number of antibody-secreting cells in the bone marrow at day 112 (Fig. 2E). Together, these data suggest that pSer conjugation to the N terminus of RBD can substantially enhance humoral responses to alum:RBD immunization.

### A stabilized RBD mutant further enhances the immunogenicity of alum:RBD vaccines

We recently developed an engineered RBD variant containing two point mutations (L452K and F490W; table S1) to improve manufacturability and stability of the antigen. This variant (hereafter, RBDJ) was also more immunogenic than Wuhan-Hu-1 RBD (hereafter, wild-type RBD) in mice (*29*). Given the inconsistent neutralizing responses observed in mice immunized with pSer_4_-RBD, we thus tested whether RBDJ would benefit from pSer tagging and assessed whether increasing the valency of the pSer tag could further enhance antibody responses. To this end, we synthesized RBDJ N-terminally modified with a pSer_4_ or pSer_8_ tag (fig. S2A). Both pSer_4_-RBDJ and pSer_8_-RBDJ adsorbed efficiently to alum, and pSer_8_-RBDJ showed slightly higher retention on alum over time on exposure to serum/phosphate buffer (fig. S2, B and C). As observed with the wild-type RBD, pSer-RBDJ protein bound to alum particles retained robust binding to hACE2, CR3022, H4, and B38 (fig. S2, D and E).

**Fig. 3.**
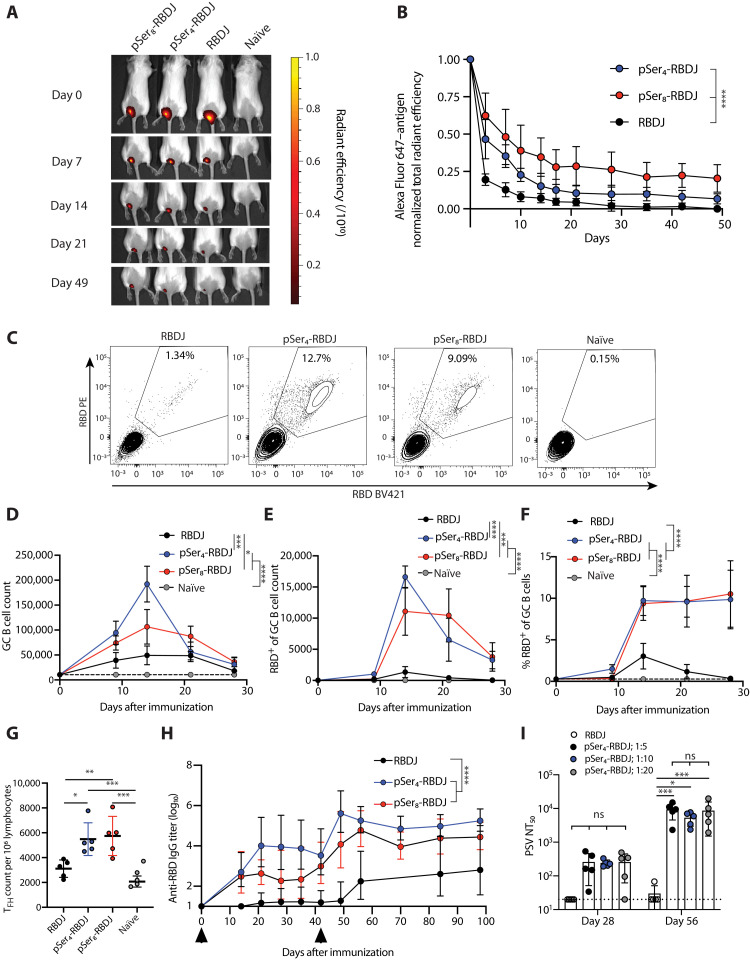
pSer-conjugated mutant RBDs elicit potent GC responses and neutralizing antibodies in mice. (**A** and **B**) Mice (*n* = 4 per group) were immunized with 10 μg of labeled unmodified or pSer-conjugated RBDJ + 100 μg of alum, and injection site fluorescence was tracked longitudinally by in vivo imaging system (IVIS) imaging. Shown are whole-animal images (A) and fluorescence quantification (B) (means ± SD). (**C** to **G**) Mice (*n* = 5 per group) were immunized, and GC and T_FH_ responses in dLNs were analyzed by flow cytometry. Shown are representative gating of RBD-specific GC B cells (C), total GC B cell counts (D), RBD-specific GC B cell counts (E), percent RBD-specific GC B cells (F), and T_FH_ enumeration at day 14 (G). Shown are means ± SEM. (**H**) Mice (*n* = 5 per group) were immunized twice (indicated by arrows), and serum antibody responses (geometric means ± geometric SD) were tracked by ELISA. (**I**) Mice (*n* = 5 per group) were immunized with varying antigen densities on alum. pSer_4_-RBDJ was loaded on alum at the indicated ratios; all groups received 200 μg of alum. Shown are PSV NT_50_; dotted line indicates LOD. Shown are means ± SD. Statistical significance was determined by two-way ANOVA followed by Tukey’s post hoc test (B and D to G) or Sidak’s multiple comparisons test (I). ns, *P* > 0.05; *P < 0.05; **P < 0.01; ***P < 0.001; ****P < 0.0001.

We previously found that pSer-modified HIV Env proteins trafficked to lymph nodes bound to alum particles, such that antigen-specific B cells directly internalized antigen-decorated alum particles (*26*). To gain insight into the behavior of pSer-tagged RBDJ and assess whether increased pSer valency affected antigen availability kinetics in vivo, we fluorescently labeled the pSer-RBDJ proteins with an Alexa Fluor 647 dye. Mice were immunized subcutaneously with these labeled vaccines near the tail base, and the kinetics of antigen clearance from the injection site over time were tracked by whole-animal fluorescence imaging (Fig. 3A). Fluorescence from the RBD antigen steadily cleared from the immunization site, with the rate of decay ordered as RBDJ > pSer_4_-RBDJ > pSer_8_-RBDJ (Fig. 3B).

To determine whether these distinct vaccine kinetics affected the immune response, we first quantified GC responses following alum:RBDJ immunization. Flow cytometry analysis of draining inguinal lymph nodes (dLNs) harvested at staggered time points after injection revealed that pSer-tagged RBDs elicited notably stronger GC responses than traditional alum:RBDJ immunization (Fig. 3, C to F, and fig. S3, A to C). The total GC response peaked at day 14, with pSer_4_-RBDJ eliciting the strongest response (Fig. 3D and fig. S3C). Even more notable was the impact on antigen-specific GC B cells: Both pSer_4_-RBDJ and pSer_8_-RBDJ primed a substantial population of RBD-specific GC B cells, while antigen-specific cells were very low in the alum:RBDJ control group across the entire time course (Fig. 3, C, E, and F, and fig. S3C). In addition, pSer-conjugated RBDJ elicited ~2-fold greater T follicular helper cell (T_FH_) responses than the unmodified immunogen (Fig. 3G). These enhanced GC and T_FH_ responses correlated with greatly increased IgG antibody responses for mice immunized with pSer-RBDJ compared to RBDJ (Fig. 3H), and these antibodies exhibited a significantly higher ability to block hACE2-RBD interactions (fig. S4A). Total binding IgG ELISA titers and hACE2 binding inhibition trended to be higher with pSer_4_-RBDJ versus pSer_8_-RBDJ, but these differences did not reach statistical significance. Notably, the antigen drainage characterization (Fig. 3, A and B) suggested that a portion of pSer_8_-RBDJ may be irreversibly trapped at the injection site. If we subtracted the plateau fluorescence signal from the total fluorescence of the pSer_8_- group over time, then the resulting “bioavailable” pSer_8_-RBDJ trajectory looks very similar to that of pSer_4_-RBDJ (fig. S4B). Furthermore, longitudinal tracking of alum drainage from the injection site revealed that ~58% of alum remains at the injection site 70 days after immunization for pSer_8_-RBDJ compared to ~37% for pSer_4_-RBDJ (fig. S4C). We hypothesize that these altered kinetics for antigen and alum clearance observed with the longer pSer_8_-tagged immunogen may reflect some level of inter-alum particle cross-linking mediated by the longer peptide tag, which inhibits disaggregation of alum particles and promotes their phagocytosis locally at the injection site, limiting delivery to the dLNs. pSer_4_-RBDJ elicited peak serum IgG titers ~4-fold greater than the same dose of pSer_4_-RBD with more consistent seroconversion (Figs. 2A and 3H). Together, these data indicate that pSer-RBDJ vaccines elicit greatly enhanced GC and serum antibody responses compared to traditional immunization with admixed RBDJ and alum.

We also investigated the impact of alum dose and antigen density on humoral immune responses. Varying the amount of RBDJ added to a fixed amount of alum, we identified a range of antigen densities for which there was comparable pSer_4_-RBDJ loading and retention on alum (fig. S5A). On the basis of the reported surface area of Alhydrogel alum (*30*), antigen:alum mass ratios of 1:5, 1:10, and 1:20 correspond to an estimated average spacing between RBDs on alum particles of 9.6, 13.5, and 19.2 nm, respectively. To determine whether antigen density variation in this range affects the immune response, we immunized mice with a constant dose of pSer_4_-RBDJ loaded on varying quantities of alum (50, 100, or 200 μg) and tracked the serum antibody responses longitudinally. Differences between these three groups were very modest and not statistically significant (fig. S5B). Examining GC responses 14 days after immunization, antigen-specific GC B cell frequencies showed a slight trend toward increased responses at lower antigen density/higher alum dose, but these differences again were not significant (fig. S5C). These experiments are potentially confounded by the convolution of antigen density with alum amount, and thus, we also devised a second experimental approach: Immunizations were prepared by first loading antigen on alum at the specified ratios and then supplementing in extra alum just before immunization to bring the alum dose to 200 μg for all mice. To confirm that pSer_4_-RBDJ would not redistribute when additional alum was added, we imaged alum particles loaded with fluorophore-tagged pSer_4_-RBDJ that were mixed with RBD-free alum tagged by a low density of pSer_4_-Alexa dye and incubated together for 2 days. As shown in fig. S5 (D and E), no transfer of pSer_4_-RBDJ (red) to the bare alum particles (cyan) was observed when the mixture was examined by microscopy. Therefore, using this approach, we repeated immunizations varying the antigen density across the same RBD:alum mass ratios and assessed GC responses, serum IgG over time, and neutralizing antibody titers. Similar to the previous experiments, GC responses were not statistically different between the groups (fig. S5F). There was a transient enhancement in humoral responses after prime with increasing antigen density, but all groups responded similarly after boost, and PSV NT_50_ values were not different across the three pSer_4_-RBDJ groups (Fig. 3I and fig. S5, G and H). The neutralizing responses elicited by pSer_4_-RBDJ:alum, however, were significantly higher than unmodified RBDJ and were notably more consistent than we observed with wild-type pSer_4_-RBD:alum, with all animals primed to produce high levels of neutralizing responses at a mean PSV NT_50_ value of ~5270 2 weeks after boost (n.b., compare 1:10 antigen density in Figs. 3I with 2C). Thus, pSer_4_ modification of RBDJ enhanced GC and neutralizing antibody responses, but these responses were not sensitive to the density of antigen loading on alum.

### Conjugation of antigen and adjuvants to alum synergistically amplifies humoral responses

**Fig. 4.**
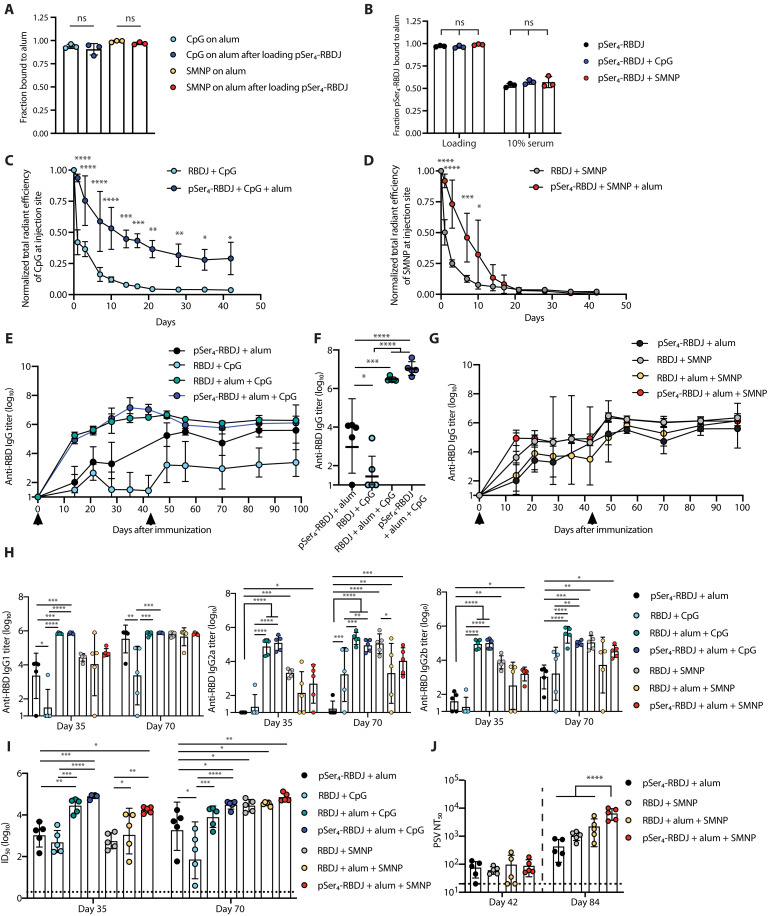
Combining pSer-RBD with alum-binding coadjuvants enhances humoral immunity. (**A**) CpG or SMNP was added to alum for 30 min, and the fraction of alum-bound adjuvant was measured. (**B**) The fraction of pSer_4_-RBDJ binding to alum coloaded with CpG or SMNP was assessed before (loading) and after 24 hours incubation (10% mouse serum at 37°C). (**C** and **D**) Mice (*n* = 3 per group) were immunized with 30 μg of labeled CpG (C) or 5 μg of labeled SMNP (D) with 10 μg of RBDJ ± 100 μg of alum, and injection site fluorescence was assessed by IVIS. (**E** to **J**) Mice (*n* = 5 per group) were immunized twice (indicated by arrows) with RBDJ combined with the indicated adjuvants. Shown are serum IgG titers over time (E and G), total IgG from individual animals on day 42 (F), antibody titers by isotype on indicated days (H), ID_50_ titer values assessed for hACE2-RBD binding in the presence of indicated sera (I), and PSV NT_50_ (J). Dotted line indicates the LOD. Shown are means ± SD (A to D and J) or geometric means ± geometric SD (E to I). Statistical significance was determined by one-way ANOVA followed by Tukey’s post hoc test (A, B, E, F, H, and I) or Sidak’s multiple comparison test (C, D, and J). ns, *P* > 0.05; *P < 0.05; **P < 0.01; ***P < 0.001; ****P < 0.0001.

Although pSer anchoring RBD to alum greatly enhanced its immunogenicity, alum remains an adjuvant with modest potency in large animals and humans. We hypothesized that combining alum with a molecular coadjuvant using the same ligand exchange reaction used to anchor RBD immunogen would synergistically enhance the immune response, by prolonging the exposure of dLNs to both antigen and inflammatory cues. We thus tested the behavior of two clinically relevant phosphate-containing coadjuvants, CpG, a single-stranded DNA TLR9 agonist containing phosphorothioates in the oligonucleotide backbone, and SMNP (*27*), an ISCOMs (immunostimulating complexes) -like, ~40-nm-diameter nanoparticle formed by the self-assembly of phospholipids, saponin, and the TLR4 agonist MPLA, which binds to alum via phosphate groups of the lipids and MPLA, in combination with pSer_4_-RBDJ and alum. Both CpG and SMNP demonstrated strong alum adsorption and retention on alum (3:10 and 1:20 mass ratios, respectively) in the presence of mouse serum, suggesting strong ligand exchange–mediated binding (Fig. 4A). We sequentially added pSer_4_-RBDJ and the two coadjuvants to alum; we found that neither CpG nor SMNP displaced bound pSer_4_-RBDJ (Fig. 4B). Whole-animal fluorescence imaging of the injection site following immunization with labeled CpG or SMNP adsorbed to alum together with pSer_4_-RBD revealed sustained drainage of the coadjuvants compared to injection of these adjuvants in the absence of alum (Fig. 4, C and D). Notably, addition of these coadjuvants did not disrupt the sustained drainage of pSer_4_-RBDJ when loaded on alum: When CpG or SMNP was adsorbed to alum with labeled pSer_4_-RBDJ, there was no significant difference in the kinetics of antigen clearance when compared to pSer_4_-RBDJ on alum alone (fig. S6A).

To investigate the impact of these alum-bound coadjuvants on humoral responses, we immunized mice with combinations of CpG or SMNP bound to alum with RBDJ or pSer_4_-RBDJ and tracked serum antibody responses over time. Notably, the addition of CpG to pSer_4_-RBDJ:alum or RBDJ:alum immunizations markedly enhanced IgG antibody titers compared to pSer_4_-RBDJ:alum or soluble RBDJ + CpG following the priming immunization (Fig. 4, E and F). There were also trends of increased IgG antibody titers for alum-bound antigen and coadjuvant SMNP (Fig. 4G). Examination of individual IgG isotypes showed that IgG1, IgG2a, and IgG2b titers were all substantially increased when pSer-RBDJ:alum was combined with each of the coadjuvants (Fig. 4H), and the IgG2a/IgG1 and IgG2b/IgG1 ratios were increased with the addition of the coadjuvants (fig. S6B). The addition of CpG and SMNP to pSer:alum immunizations also elicited more functional antibody responses, as serum from immunized mice demonstrated stronger inhibition of hACE2-RBD binding both after prime and after boost (Fig. 4I). Notably, maximal hACE2 binding inhibition/neutralizing responses required that alum was combined with one of the coadjuvants and that the RBD was pSer-modified. This finding was even more starkly illustrated by PSV neutralizing antibody titers measured for animals immunized with pSer-RBDJ:alum ± SMNP: Immunization with pSer_4_-RBDJ:alum or RBDJ + SMNP elicited PSV NT_50_ titers ~10-fold weaker than the pSer_4_-RBDJ:alum:SMNP combination (Fig. 4J and fig. S6C). Notably, there was no statistically significant correlation between serum IgG ELISA binding titers and PSV NT_50_ at day 42 or 84 (fig. S6D), suggesting that the serum IgG ELISA binding titers are not predictive of neutralizing titers for these groups.

To further investigate the basis of this enhanced neutralizing antibody response, we investigated the impact of CpG or SMNP coadjuvants on the cellular localization of antigen. Mice were immunized with Alexa Fluor–labeled RBDJ, and the number of cells positive for antigen was assessed among B cells, monocytes, neutrophils, subcapsular sinus macrophages, medullary macrophages, and dendritic cells (fig. S7, A and B). B cells showed a significant increase in antigen uptake following pSer_4_-RBDJ:alum + SMNP immunization compared to RBDJ:alum and pSer_4_-RBDJ:alum, whereas there was a significant increase in monocyte uptake of antigen for pSer_4_-RBDJ:alum + CpG compared to RBDJ:alum and pSer_4_-RBDJ:alum. These differences in antigen distribution may contribute to the synergistic benefit of coadjuvants with pSer_4_-RBDJ:alum. To elucidate the basis of the enhanced neutralization responses in mice immunized with pSer_4_-RBDJ:alum + SMNP despite comparable overall IgG titers with RBDJ + SMNP, we immunized mice with unmodified RBDJ or pSer_4_-RBDJ and alum and/or SMNP and assessed the RBD-specific GC B cell responses in the dLNs at day 14 after immunization. Notably, pSer_4_-RBDJ:alum + SMNP elicited significantly higher RBD-specific GC B cell responses compared to RBDJ + SMNP (fig. S7C). These data suggest that stronger neutralizing responses observed in mice immunized with pSer_4_-RBDJ:alum + SMNP compared to RBDJ + SMNP are driven by more robust antigen-specific GC responses when alum anchoring and SMNP are combined. Hence, co-conjugation of molecular adjuvants and the immunogen with alum synergistically amplifies humoral immunity to RBD.

### Sustained antigen and coadjuvant drainage elicits strong responses in RMs

**Fig. 5.**
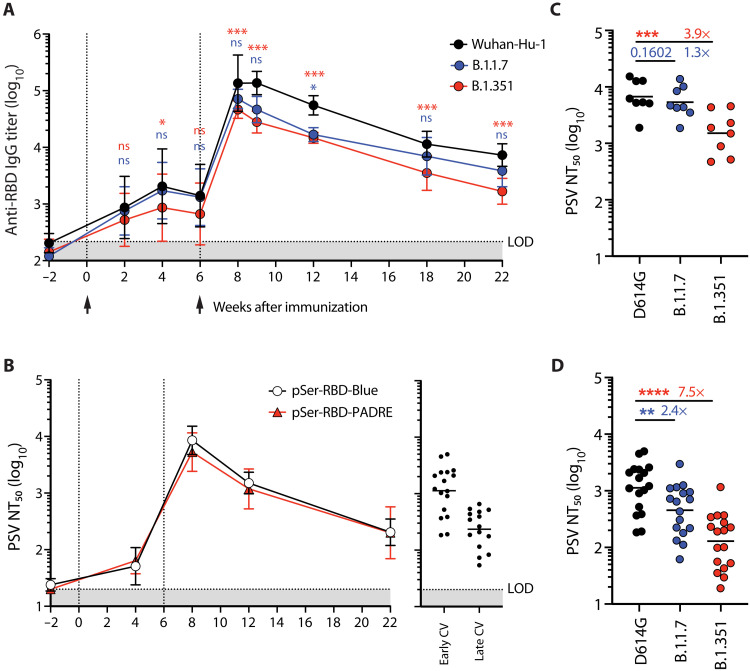
pSer-RBDJ:alum:SMNP immunization generates robust antibody titers against circulating SARS-CoV-2 variants and neutralizing antibody titers in RMs. RMs were immunized with 50 μg of pSer-RBDJ-Blue (*n* = 4 animals) or 50 μg of pSer-RBDJ-PADRE (*n* = 4 animals) combined with 0.5 mg of alum and 50 μg of SMNP subcutaneously in the left and right deltoid at weeks 0 and 6. Sera were collected over the course of the immune response. (**A**) RBD-specific serum IgG titers detected by ELISA against Wuhan-Hu-1, B.1.1.7, and B.1.351 RBD variants (grouped from all eight animals). Values plotted are geometric means ± geometric SD. Arrows indicate immunization time points. (**B**) Serum PSV NT_50_ to SARS-CoV-2 D614G over the course of the immunization. Arrows indicate immunization time points. Statistical significance between groups receiving the two immunogens was determined by Mann-Whitney test. Neutralization titers of convalescent (CV) human serum samples are shown on the right for comparison. The *y* axes of the two graphs are identical. Early convalescent samples are from an average of 32.6 days after symptom onset (range, 21 to 43 days; median, 33 days), and late convalescent samples are from an average of 178.7 days after symptom onset (range, 151 to 221 days; median, 179). (**C**) Serum PSV NT_50_ at week 8 after immunization to SARS-CoV-2 D614G, B.1.1.7, and B.1.351 PSVs. Geometric mean titer is shown by the black line. (**D**) Early convalescent serum neutralization titers to D614G, B.1.1.7, and B.1.351 PSVs. Reduction in average ID_50_ titers is shown in numbers. Friedman test followed by Dunn’s multiple comparisons test was conducted to compare serum binding or neutralization titers to B.1.1.7 and B.1.351 isolates relative to the D614G control. LOD indicates the lowest serum dilution tested. ns, *P* > 0.05; *P < 0.05; **P < 0.01; ***P < 0.001; ****P < 0.0001.

On the basis of the strong neutralization responses elicited in mice by pSer_4_-RBDJ:alum with added SMNP, we evaluated this immunization in a pilot study in RMs. Although CpG was potent as a coadjuvant in mice, we chose to evaluate SMNP in macaques due to species-based differences in the expression of TLR9: While TLR9 is broadly expressed by B cells, myeloid cells, and dendritic cells in mice, expression is restricted to plasmacytoid dendritic cells and B cells in RMs and humans (*31*). Likely related to this issue, doses and potency of CpG in NHPs vary widely in published studies. Because of these issues, we opted instead to use SMNP, which has performed reliably well in several studies in RMs (*27*). In an attempt to ensure sufficient T cell help in the RMs, we designed RBDJ variants incorporating helper epitopes derived from the SARS-CoV-2 S protein (an epitope we refer to as “Blue”) (*32*) or the “universal” helper epitope PADRE (table S1) (*33*–*35*). These constructs exhibited the expected loading and retention behavior on alum in the presence of mouse and RM serum and preserved antigenicity when bound to alum (fig. S8). Two groups of animals were immunized with the constructs, and antibody responses were assessed longitudinally. These immunizations elicited high levels of serum IgG binding titers against the SARS-CoV-2 Wuhan-Hu-1 RBD; these sera also recognized the circulating SARS-CoV-2 variants of concern, B.1.1.7 (alpha, UK) and B.1.351 (beta, South Africa), albeit with binding titers ~3- and ~5-fold lower on average at peak (week 8), respectively (Fig. 5A). Animals had low neutralizing antibody responses to the autologous strain after one immunization but generated high PSV NT_50_ value following a single boost. These titers remained greater than or comparable to neutralizing responses in human convalescent sera through 22 weeks after vaccination (Fig. 5B). PSV NT_50_ value peaked ~8-fold higher in immunized animals than in COVID-19 cases (mean, 8068.3; mean, 1129; Fig. 5B). There were no differences in the magnitude of neutralizing antibody responses to either of the pSer_4_-RBDJ immunogens tested. Serum from immunized animals retained comparable neutralizing activity to the B.1.1.7 strain and had a ~4-fold reduction in the neutralization of the B.1.351 variant (Fig. 5C and fig. S9A). Notably, the neutralization by the immunized animals was less sensitive to variant changes than neutralization observed for human COVID-19 convalescent sera we evaluated, which had ~2.4- and ~7.5-fold loss of potency against these two common variants (Fig. 5D and fig. S9, B and C).

## DISCUSSION

There is a need for additional safe and effective SARS-CoV-2 vaccines to facilitate global vaccine coverage. Given the emergence of novel SARS-CoV-2 variants, it is especially important that these vaccines elicit responses that retain activity against circulating variants of concern. Subunit vaccines are an attractive approach to achieving global coverage, as they can be rapidly scaled for manufacturing, and their distribution does not require ultracold storage temperatures. Here, we describe an approach using alum, a low-cost adjuvant with widespread clinical use, that elicited potent humoral immune responses and neutralization in mice and RMs against SARS-CoV-2. By modifying the RBD antigen with a short peptide linker, the duration of antigen drainage from the injection site was substantially extended, leading to strong antigen-specific GC responses that lasted more than a month after immunization. Through the optimization of this immunization platform, testing the impact of N- versus C-terminal pSer conjugation, pSer valency, antigen density, and the addition of alum-binding coadjuvants, the platform achieved continually higher and more consistent antibody and neutralization responses in mice (fig. S10). Notably, the addition of CpG or SMNP coadjuvants to pSer-RBD + alum immunizations also promoted a more balanced T helper cell 1 (T_H_1)/T_H_2 bias to the antibody response.

In the context of HIV vaccine development, previous work has investigated the impact of “extended dosing” approaches on humoral immune responses to subunit vaccines. Prolonged antigen availability over 1 to 2 weeks facilitated by repeated dosing, microneedle patches, or osmotic pumps has been shown to amplify humoral immune responses compared to conventional bolus immunization (*36*–*38*). These approaches were shown to enhance and prolong GC B cell responses and T_FH_ responses. The pSer modification approach used here provides a simple and robust strategy to prolong antigen availability in a clinically translatable vaccine regimen. The alum-anchoring strategy used here has the additional capacity to help potentiate B cell responses by presenting many copies of antigen bound to a single alum particle, promoting B cell receptor cross-linking and early signaling/B cell activation (*26*). However, in the case of RBD, varying antigen density did not affect any of the measures of the humoral responses assessed here, suggesting either that the RBD densities explored here did not cover a wide enough range to detect an effect on B cell triggering or that some release of pSer-RBD from alum particles occurs over time, thus diluting the “alum presentation” effect.

Studies applying repeated injections to achieve extended dosing in cancer vaccines have demonstrated the importance of sustained exposure to both antigen and inflammatory cues in peptide vaccines for optimal T cell responses (*39*), but the role of extended adjuvant exposure on humoral immunity is not well understood. To couple the kinetics of antigen and adjuvant delivery to lymph nodes, we tested here the use of two different molecular adjuvants, CpG and a nanoparticle-formulated saponin, each of which could undergo the same type of ligand exchange reaction with alum as used in our pSer-modified immunogens. With each of these coadjuvants, we observed sustained release from the injection site in the presence of alum. These altered vaccine kinetics correlated with enhanced antibody responses and neutralization that were much more than additive over the individual responses elicited by alum or the coadjuvants in isolation, suggesting strong synergy induced by alum binding. These findings are in concordance with prior studies reporting synergy between alum and CpG in promoting strong humoral and cellular immune responses (*40*–*43*). A recent phase 1 clinical trial of a SARS-CoV-2 S protein combined with alum/CpG has reported promising safety and immunogenicity data (*44*). We hypothesize that altered delivery kinetics achieved by ligand exchange binding to alum play an important role in the potency of this adjuvant combination.

Two immunizations combining pSer-modified RBD immunogen with alum and SMNP elicited peak mean PSV NT_50_ value of ~8000 in RMs. Despite the challenge in comparing neutralizing antibody assays from laboratory to laboratory, these results appear competitive with published SARS-CoV-2 vaccine responses in NHPs (*8*, *45*–*50*), including a nanoparticle subunit vaccine (*51*, *52*). A study of recombinant RBD combined with traditional alum immunization reported NT_50_ value of only ~100 at a similar time point after boost (*10*). Strong neutralizing responses were elicited herein not only to the SARS-CoV-2 D614G viral strain but also against two circulating SARS-CoV-2 variants of concern, B.1.1.7 and B.1.351. Few studies to date have examined the durability of neutralizing responses elicited against SARS-CoV-2 in NHPs. Some protein-in-adjuvant vaccines are known to elicit serum antibody binding and neutralization titers that decay ~10-fold over 2 to 3 months after booster immunization and then plateau at relatively sustained levels (*53*, *54*). However, in this study, at 22 weeks after the primary pSer-RBD:alum:SMNP immunization, serum SARS-CoV-2 neutralizing titers remained comparable to titers measured in convalescent patient sera. Together these findings suggest that the combination of pSer-mediated immunogen binding to alum with SMNP coadjuvant delivery is an effective adjuvant combination both in mice and NHPs.

As a platform, this technology promotes sustained antigen and coadjuvant drainage from the injection site, inducing potent humoral immune responses in mice and NHPs against SARS-CoV-2 using alum, a low-cost adjuvant with widespread clinical use. In the context of more immunogenic antigens, this platform could also be beneficial to promotion of a dose-sparing strategy to increase vaccine availability. There has been great interest in combining adjuvants to steer immune responses and promote synergistic effects (*55*). Our findings offer further support for the idea that combinations of adjuvants can enable new immunological mechanisms of action, providing vaccine formulations with activity greater than the individual components, and enhance the potency of subunit vaccine antigens.

## MATERIALS AND METHODS

### pSer peptide synthesis

pSer peptide linkers were synthesized using solid-phase synthesis on low-loading TentaGel Rink Amide resin (0.2 meq/g; Peptides International, catalog no. R28023) as described previously (*26*). Briefly, resin was deprotected with 20% piperidine (Sigma-Aldrich, catalog no. 411027) in dimethylformamide (DMF; Sigma-Aldrich, catalog no. 319937-4L), and peptide couplings were performed with 4 eq of Fmoc-Ser(PO(OBzl)OH)-OH (MilliporeSigma, catalog no. 8520690005) and 3.95 equivalents of hexafluorophosphate azabenzotriazole tetramethyl uranium (MilliporeSigma, catalog no. 148893-10-1) for 2 hours at 25°C. pSer residues were deprotected with 5% 1,8-diazabicyclo[5.4.0]undec-7-ene (Sigma-Aldrich, catalog no. 139009) in DMF. Double couplings were performed after the third residue. An Fmoc-protected six-unit oligoethylene glycol linker (Peptides International, catalog no. DPG-5750) was then coupled to the peptide and subsequently deprotected and reacted with *N*-maleoyl-β-alanine (Sigma-Aldrich, catalog no. 394815). Completion of each deprotection and coupling step was confirmed by a ninhydrin test (Sigma-Aldrich, catalog no. 60017). pSer side chains were deprotected, and the peptide was cleaved from the resin in 95% trifluoroacetic acid (Sigma-Aldrich, catalog no. T6508), 2.5% H_2_O, and 2.5% triisopropylsilane (Sigma-Aldrich, catalog no. 233781) for 2.5 hours at 25°C. The product was precipitated in 4°C diethyl ether (Sigma-Aldrich, catalog no. 673811) and dried under N_2_ then purified by high-performance liquid chromatography (HPLC) on a C18 column (Agilent Zorbax 300SB-C18) using 0.1 M triethylammonium acetate buffer (Glen Research, catalog no. 60-4110-62) in an acetonitrile gradient. The peptide mass was confirmed by matrix-assisted laser desorption/ionization–time-of-flight mass spectrometry. For imaging experiments, the pSer_4_–Alexa Fluor 488 conjugate was synthesized as described for the pSer component of the linker, followed by deprotection and coupling to Fmoc-5-azido-pentanoic acid (Anaspec, catalog no. AS-65518-1). The peptide was deprotected with 20% piperidine in DMF before cleavage from the resin in 95% trifluoroacetic acid, 2.5% H_2_O, and 2.5% triisopropylsilane for 2.5 hours at 25°C. The product was then precipitated in 4°C diethyl ether, dried under N_2_, and purified by HPLC on a C18 column using 0.1 M triethylammonium acetate buffer in an acetonitrile gradient. The peptide mass was confirmed by matrix-assisted laser desorption/ionization–time-of-flight mass spectrometry. This pSer_4_-azide linker was reacted with 1 equivalent of Alexa Fluor 488–dibenzocyclooctyne (DBCO) (Click Chemistry Tools, catalog no. 1278) overnight at 4°C in a Cu-free click reaction in phosphate-buffered saline (PBS) (pH 7.2 to pH 7.4) and subsequently purified by HPLC on a C18 column using 0.1 M triethylammonium acetate buffer in an acetonitrile gradient.

### Antigen production and pSer conjugation

RBD immunogens were expressed in yeast strains derived from *Komagataella phaffii* (NRRL Y-11430) as described previously (*29*). Protein was purified using the InSCyT purification module as described previously (*56*). Columns were equilibrated in buffer before each run. His-tagged RBDs were purified with a 1-ml HisTrap HP column (Cytiva Life Sciences, catalog no. 29051021) on an ÄKTA pure 25-L FPLC system (Cytiva Life Sciences, catalog no. 29018224). The column was equilibrated with a binding buffer composed of 25 mM imidazole, 25 mM sodium phosphate, and 500 mM NaCl (pH 7.4). Protein-containing supernatant was applied to the column via a S9 sample pump (Cytiva Life Sciences, catalog no. 29027745) at a rate of 2 ml/min. After washing the column with binding buffer, the his-tagged RBD (amino acids 332 to 532 of SARS-CoV-2 Wuhan-Hu-1 S protein; GenBank: MN908947.3) was eluted with 500 mM imidazole, 25 mM sodium phosphate, and 500 mM NaCl (pH 7.4). For non–his-tagged RBDs, protein-containing supernatant was adjusted to pH 4.5 using 100 mM citric acid and subsequently loaded into a prepacked 5-ml CMM HyperCel column (Pall Corporation, catalog no. PRCCMMHCEL5ML), reequilibrated with 20 mM sodium citrate (pH 5.0), washed with 20 mM sodium phosphate (pH 5.8), and eluted with 20 mM sodium phosphate (pH 8.0) and 150 mM NaCl. Eluate from column 1 above 15 milli–absorbance units (mAU) was flowed through a 1-ml prepacked HyperCel STAR AX column (Pall Corporation, catalog no. PRCSTARAX1ML). Flow-through from column 2 above 15 mAU was collected.

Antigens expressed with a free terminal cysteine were reduced at 1 mg/ml with 2 molar equivalents of tris(2-carboxyethyl)phosphine (TCEP; Thermo Fisher Scientific, catalog no. 20490) and incubated at 25°C for 10 min. TCEP was subsequently removed from reduced protein solutions using Amicon Ultra centrifugal filters [10-kDa molecular weight cutoff (MWCO); MilliporeSigma, catalog no. UFC501096] in TBS (Sigma-Aldrich, catalog no. T5912), and antigen (1 mg/ml) was reacted with 2 molar equivalents of pSer-maleimide linkers for 16 hours at 4°C in TBS (pH 7.2 to pH 7.4). Free pSer linker was subsequently removed using centrifugal filters in TBS, and pSer-antigen was buffer exchanged to PBS. The pSer_4_–cytochrome C used for antigenicity profiling of immunogens was prepared as described, using cytochrome C from *Saccharomyces cerevisiae* (Sigma-Aldrich, catalog no. C2436). The number of pSer residues conjugated to the antigen was assessed using the Malachite Green Phosphoprotein Phosphate Estimation Assay Kit (Thermo Fisher Scientific, catalog no. 23270) against a standard curve of pSer-maleimide linker. Signal from pSer-antigen was compared to the background from an unconjugated antigen control. Fluorescently labeled protein used in imaging experiments was prepared by reacting antigen (1 mg/ml) in 50 mM sodium bicarbonate buffer for 1 hour at 25°C with 6 molar equivalents of Alexa Fluor 647 *N*-hydroxysuccinimide (NHS) ester (Invitrogen, catalog no. A20006) for alum-binding studies and whole-mouse imaging or Alexa Fluor 555 NHS ester (Invitrogen, catalog no. A20009) for microscopy experiments. Labeled antigen was purified by centrifugal filtration.

### SMNP adjuvant synthesis

SMNP adjuvant was prepared as previously described (*27*). Briefly, solutions at 20 mg/ml were prepared of cholesterol (Avanti Polar Lipids, catalog no. 700000), dipalmitoylphosphatidylcholine (DPPC; Avanti Polar Lipids, catalog no. 850355), and PHAD MPLA (Avanti Polar Lipids, catalog no. 699800P) in 20% N-decanoyl-N-methylglucamine (MEGA-10) (Sigma-Aldrich, catalog no. D6277) detergent. Quil-A saponin (InvivoGen, catalog no. vac-quil) was dissolved in Milli-Q water at a final concentration of 100 mg/ml. These were mixed at a mass ratio of 10:2:1:1 (Quil-A:chol:DPPC:MPLA) and diluted in PBS to a final cholesterol concentration of 1 mg/ml. The solution was equilibrated overnight at 25°C and then dialyzed against PBS using a 10-kDa MWCO cassette. The adjuvant was then sterile filtered, concentrated using Amicon Ultra centrifugal filters (50-kDa MWCO; MilliporeSigma, catalog no. UFC505096), and purified by FPLC using a Sephacryl S-500 HR size exclusion column. SMNP labeled with Cy7 was prepared as described incorporating 1,2-distearoyl-*sn*-glycero-3-phosphoethanolamine-*N*-(Cyanine 7) (Avanti Polar Lipids, catalog no. 810347) in place of 10 mole percent of the MPLA.

### Antigen and adjuvant alum binding and release

Alexa Fluor 647–labeled antigen was loaded onto Alhydrogel (alum; InvivoGen, catalog no. vac-alu-250) in TBS at a 1:10 antigen:alum mass ratio, unless otherwise specified, for 30 min on a tube rotator at 25°C. To assess antigen binding to alum, samples were immediately centrifuged at 10,000*g* for 10 min to pellet alum, and the fluorescence of the supernatant was measured against a standard curve of labeled antigen. To assess the release of antigen from alum, mouse serum was added to antigen-alum solutions after loading to a final mouse serum concentration of 10 v/v and incubated at 37°C for 24 hours, unless otherwise specified. Samples were subsequently centrifuged at 10,000*g* for 10 min to pellet alum, and the fraction of protein bound to alum was measured by fluorescence using a Tecan Infinite M200 Pro plate reader. Experiments investigating CpG binding and release from alum were performed using fluorescein isothiocyanate (FITC)–labeled CpG 1826 (InvivoGen, catalog no. tlrl-1826f) with a 3:10 CpG:alum mass ratio. Experiments investigating SMNP binding and release from alum were performed using Cy7-labeled SMNP with a 1:20 SMNP:alum mass ratio.

### Antigenicity profiling of RBD immunogens

Antigenicity profiling of antigens was completed by comparing antibody binding curves of pSer-conjugated RBD or RBDJ on alum against those of unmodified RBD or RBDJ. To capture alum on Nunc MaxiSorp ELISA plates (Invitrogen, catalog no. 44-2404-21), plates were first coated with pSer_4_-conjugated cytochrome C at 2 μg/ml for 4 hours at 25°C. Alum was then added at 200 μg/ml and captured by pSer_4_–cytochrome C overnight at 4°C. To capture unmodified RBD, plates were coated with a rabbit anti–his-tag antibody (GenScript, catalog no. A00174-40) at 2 μg/ml overnight at 4°C. Plates were washed with 0.05% Tween 20 in PBS and incubated with protein (2 μg/ml) in 2% bovine serum albumin (BSA) in PBS for 2 hours at 25°C. CR3022 monoclonal antibody (Abcam, catalog no. ab273073), hACE2-Fc chimera (InvivoGen, catalog no. fc-hace2), H4 (InvivoGen, catalog no. cov2rbdc1-mab1), or B38 (InvivoGen, catalog no. cov2rbdc2-mab1) was added at 5 μg/ml with 1:4 serial dilutions for 2 hours at 25°C. Plates were washed, and antibody binding was detected with a goat anti-human horseradish peroxidase (HRP)–conjugated secondary antibody (Bio-Rad, catalog no. 1721050) at 1:5000 dilution in PBS containing 2% BSA and then developed with 3,3′,5,5′-tetramethylbenzidine (Thermo Fisher Scientific, catalog no. 34028), stopped with 2 N of sulfuric acid, and immediately read (450 nm with 540-nm reference) on a BioTek Synergy2 plate reader.

### Animals and immunizations

Experiments and handling of mice were conducted under federal, state, and local guidelines under an Institutional Animal Care and Use Committee (IACUC)–approved protocol. Six- to 8-week-old female BALB/c mice were purchased from the Jackson Laboratory (stock no. 000651). Immunizations were prepared by mixing 10 μg of antigen and 100 μg of alum in 100 μl of sterile TBS (Sigma-Aldrich, catalog no. T5912) per mouse unless otherwise specified. Antigen was loaded onto alum for 30 min on a tube rotator before immunization. When CpG 1826 or SMNP was added into the immunization, antigen was first loaded onto alum for 30 min on a rotator, after which 30 μg of CpG 1826 or 5 μg of SMNP was added into the immunization and incubated with antigen-alum formulations for 30 min before immunization. This dose of SMNP corresponds to 5 μg of Quil-A and 0.5 μg of MPLA. Experiments in which antigen density was altered but the total alum dose remained the same, antigen was loaded onto alum at the indicated antigen:alum mass ratio for 30 min and supplemented alum added just before immunization to bring the total alum dose to 200 μg per mouse. Mice were immunized subcutaneously at the tail base with 50 μl on each side of the tail base and were subsequently boosted 6 weeks after prime.

This study used eight Indian-origin RMs housed at Yerkes National Primate Research Center, an animal care facility accredited by the U.S. Department of Agriculture and the Association for Assessment and Accreditation of Laboratory Animal Care International. All procedures were approved by the Emory University IACUC. Immunizations for RMs were prepared by first mixing 100 μg of antigen and 1000 μg of alum in TBS for 30 min and then adding 100 μg of SMNP, per animal. Animals were immunized subcutaneously with the doses split between the left and right deltoid and boosted at 6 weeks after prime. RMs used in this study were shared with a separate ongoing study assessing responses to an HIV Env-antigen, immunized at a different site.

### Antigen-binding ELISA

Serum was collected from mice retro-orbitally using capillary tubes and stored at −20°C until analysis. To determine serum IgG titers with RBD, Nunc MaxiSorp plates (Invitrogen, catalog no. 44-2404-21) were coated with a rabbit anti–his-tag antibody (GenScript, catalog no. A00174-40) at 2 μg/ml for 4 hours at 25°C in PBS and blocked with 2% BSA in PBS overnight at 4°C. Plates were washed with 0.05% Tween 20 in PBS, and RBD was added at 2 μg/ml in 2% BSA in PBS for 2 hours. Serum dilutions (1:10 dilution followed by 1:50 dilution with 1:4 serial dilutions) were incubated in the plate for 2 hours. Plates are washed again, incubated with a goat anti-mouse IgG HRP-conjugated secondary (Bio-Rad, catalog no. 1721011) at 1:5000 dilution, then developed with 3,3′,5,5′-tetramethytlbenzidine (Thermo Fisher Scientific, catalog no. 34028), stopped with 2 N of sulfuric acid, and immediately read (450 nm with 540 nm reference) on a BioTek Synergy2 plate reader. To determine serum IgG titers for mice immunized with RBDJ, protein was coated directly on Corning Costar high-binding 96-well plates (catalog no. 9018/3690) at 2 μg/ml in PBS overnight at 4°C and blocked for 2 hours and subsequently followed the protocol for RBD ELISAs. Isotype ELISAs followed the same protocol but used goat anti-mouse IgG1 HRP cross-adsorbed secondary antibody (Invitrogen, catalog no. A10551), goat anti-mouse IgG2a HRP cross-adsorbed secondary antibody (Invitrogen, catalog no. M32207), or goat anti-mouse IgG2b cross-adsorbed secondary antibody (Invitrogen, catalog no. M32407) at 1:2000 dilution.

For ELISAs against SARS-CoV-2 variants, Corning Costar high-binding 96-well plates (Corning, catalog no. 3690) were directly coated overnight at 4°C with 1 μg/ml of the RBD variant of interest. The RBD variants with the N501Y mutation (B.1.1.7) and K417N_E484K_N501Y mutations (B.1.315) were obtained from GenScript (GenScript, catalog nos. Z0353 and Z03537, respectively). Coated plates were washed 5× with wash buffer [PBS (pH 7.4) and 0.05% (v/v) Tween 20]. Plates were blocked for 1 hour at room temperature with blocking buffer [PBS (pH 7.4) and 3% (w/v) BSA]. Heat-inactivated NHP serum was serially diluted in dilution buffer [PBS (pH 7.4) and 1% (v/v) BSA] and added to the plates for 1 hour at 25°C. Plates were washed 5× with wash buffer, after which goat anti-rhesus IgG-HRP secondary antibody (SouthernBiotech, catalog no. 6200-05) diluted in dilution buffer was added for 1 hour at 25°C. Plates were washed 5× for a final time with wash buffer and developed with 1-Step Ultra TMB-ELISA (Thermo Fisher Scientific, catalog no. 34029). Reaction was stopped with 2 N of sulfuric acid (Ricca Chemical Company, catalog no. 8310-32). Signal was read at optical density at 450 nm on an EnVision plate reader (PerkinElmer). Recombinantly expressed CR3022 monoclonal antibody (mAb) was run on one lane of each plate for normalization. For wells in which CR3022 was added as the primary antibody, peroxidase-conjugated donkey anti-human IgG (Jackson ImmunoResearch, catalog no. 709-035-149) was used as the secondary antibody.

### ACE2 competition ELISA

Surrogate virus neutralization ELISAs (GenScript, catalog no. L00847A) were performed following the manufacturer’s protocol. Briefly, mouse serum samples were diluted at 1:10 with 1:3 serial dilutions and mixed 1:1 with RBD-HRP for 30 min at 37°C. Samples were then added to hACE2-coated plates and incubated for 15 min at 37°C. Plates were developed for 15 min with 3,3′,5,5′-tetramethytlbenzidine and stopped with 1 N of sulfuric acid, and the absorbance at 450 nm was immediately read on a BioTek Synergy2 plate reader. ID_50_ values were calculated using a nonlinear fit of individual dilution curves.

### PSV neutralization analysis

To assess neutralization in mouse serum samples, SARS-CoV-2 PSVs expressing a luciferase reporter gene were generated similar to an approach described previously (*57*, *58*). Briefly, human embryonic kidney (HEK) 293T cells were cotransfected with the packaging plasmid psPAX2 (AIDS Resource and Reagent Program), luciferase reporter plasmid pLenti-CMV Puro-Luc (Addgene, catalog no. 17477), and S protein expressing pcDNA3.1-SARS CoV-2 SΔCT using Lipofectamine 2000 (Thermo Fisher Scientific, catalog no. 11668030). Pseudo-type viruses were collected from culture supernatants 48 hours after transfection and purified by centrifugation and 0.45-μm filtration. To assess the neutralization activity of the mouse serum samples, serum was inactivated at 56°C for 30 min. HEK293T-hACE2 cells were seeded overnight in 96-well tissue culture plates at a density of 1.75 × 10^4^ cells per well. Threefold serial dilutions of heat-inactivated serum samples were prepared and mixed with 50 μl of PSV, followed by incubation at 37°C for 1 hour before adding the mixture to HEK293T-hACE2 cells. After incubation for 48 hours, cells were lysed using Steady-Glo Luciferase Assay (Promega, catalog no. E2510) according to the manufacturer’s instructions. SARS-CoV-2 PSV neutralization titers were defined as the sample dilution at which a 50% reduction in relative light unit was observed relative to the average virus control wells.

PSV neutralization assays of RM and convalescent samples were performed as described previously (*59*). Briefly, Vero cells (American Type Culture Collection, catalog no. CCL-81) were seeded into Corning 96-well clear flat-bottom plates (Corning, catalog no. 3603) the morning of the assay at 2.5 × 10^4^ cells per well. Recombinant SARS-CoV-2 S protein (GenBank: QHD43416.1) with D614G, S-B.1.1.7 (69–70 deletion, 144 deletion, N501Y, A570D, D614G, and P681H), and S-B.1.351 (L18F, D80A, and D215G, 241–243 deletion, K417N, E484K, N501Y, D614G, and A71V) pseudo-typed VSV-DG-GFP was incubated with serially diluted heat-inactivated RM sera or convalescent plasma for 1 to 1.5 hours at 37°C. The virus was then allowed to infect Vero cells for ~16 hours at 37°C in 5% CO_2_, after which virus was removed and fixed in PBS (pH 7.4), 4% (v/v) formaldehyde (Polysciences Inc., catalog no. 04018), and Hoescht (1 μg/ml; Thermo Fisher Scientific, catalog no. 62249). Cells were imaged on a CellInsight CX5 imager, and the total number of cells and infected GFP-expressing cells were counted to determine percent inhibition. Serum neutralization ID_50_ titers were calculated using the One-Site Fit Log IC_50_ (median inhibitory concentration) model in Prism 8.0 (GraphPad). The curve fit was constrained to 100% inhibition for a few weakly neutralizing serum samples that exhibited just beyond 50% but not near complete neutralization at the lowest dilution. Samples that did not reach 50% inhibition at the lowest serum dilution of 1:20 were considered non-neutralizing. The average ID_50_ titers calculated from two to four independent replicates were plotted.

### Enzyme-linked immune absorbent spot analysis

Bone marrow enzyme-linked immune absorbent spots (ELISpots) were performed in mice 16 weeks after prime following the manufacturer’s protocol (MabTech, catalog no. 3825-2A) unless otherwise specified. Briefly, 96-well polyvinylidene difluoride ELISpot plates (MilliporeSigma, catalog no. MSIPS4510) were treated with 35% ethanol before coating with anti-mouse IgG at 15 μg/ml in sterile PBS overnight at 4°C. Cells were isolated from the femur and tibia of mice, ACK lysed, and 70-μm filtered in complete media [RPMI 1640 containing 10% fetal bovine serum, penicillin-streptomycin (100 U/ml), and 1 mM sodium pyruvate]. The next day, plates were blocked with complete media for at least 30 min before adding cells with three technical replicates per mouse. For total IgG and antigen-specific IgG, 100,000 and 500,000 cells were added per well, respectively, and incubated at 37°C with 5% CO_2_ for 16 hours. Plates were then washed with PBS. Antigen-specific responses were determined by adding biotinylated RBD (1 μg/ml) in PBS with 0.5% BSA to each well for 2 hours at 25°C. Total IgG responses were determined by adding anti-mouse IgG-biotin detection antibody (1 μg/ml) in PBS with 0.5% BSA to each well for 2 hours at 25°C. Plates were washed again in PBS and incubated with 1:1000 streptavidin–alkaline phosphatase in PBS with 0.5% BSA for 1 hour at 25°C. After washing, plates were developed with bromochloroindolyl phosphate–nitro blue tetrazolium substrate (MabTech, catalog no. 3650-10), developed for 20 min, quenched with H_2_O, and dried before quantification on an ImmunoSpot CTL analyzer.

### GC and T_FH_ responses

The inguinal lymph nodes were collected from immunized mice 14 days after immunization unless otherwise specified. For GC analysis, cells were stained for viability (Thermo Fisher Scientific Live/Dead Fixable Aqua, catalog no. L34957) and against CD3e (BV711, 145-2C11 clone; BioLegend, 100349), B220 (PE-Cy7, RA3-6B2 clone; BioLegend, catalog no. 103221), CD38 (FITC, 90 clone; BioLegend, catalog no. 102705), and GL7 (PerCP-Cy5.5, GL7 clone; BioLegend, catalog no. 144609), with antigen-specific staining completed using biotinylated RBD conjugated to streptavidin-BV421 (BioLegend, catalog no. 405226) and streptavidin-PE (phycoerythrin) (BioLegend, catalog no. 405203). For T_FH_ analysis, cells were stained for viability (Live/Dead Fixable Aqua; Thermo Fisher Scientific, catalog no. L34957) and against B220 (BV510, RA3-6B2 clone; BioLegend, catalog no. 103247), CD4 (FITC, GK1.5 clone; BioLegend, catalog no. 100405), CD44 (PE-Cy7, IM7 clone; BioLegend, catalog no. 103029), PD-1 (BV421, RMP1-30 clone; BD Biosciences, catalog no. 748268), and CXCR5 (PE, 2G8 clone; BD Biosciences, catalog no. 551960). Samples were analyzed by flow cytometry on a BD Celesta and analyzed on FlowJo.

### Cellular uptake of antigen

Mice were immunized with 10 μg of Alexa Fluor 555–labeled antigen and 100 μg of alum and 5 μg of SMNP or 30 μg of CpG, and the inguinal lymph nodes were collected 7 days after immunization. Cells were stained for viability (Live/Dead Fixable Near-IR; Thermo Fisher Scientific, catalog no. L34975) and against CD3 (APC-Cy7, 17A2 clone; BioLegend, catalog no. 100221), NK1.1 (APC-Cy7, PK136 clone; BioLegend, catalog no. 108723), CD19 (PE-Cy7, 6D5 clone; BioLegend, catalog no. 115519), CD11b (BUV805, M1/70 clone; BD Biosciences, catalog no. 741934), CD11c (BUV496, HL3 clone; BD Biosciences, catalog no. 750483), Ly6C (BV650, HK1.4 clone; BioLegend, catalog no. 128049), Ly6G (BUV563, 1A8 clone; BD Biosciences, catalog no. 612921), F4/80 (BUV737, T45-2342 clone; BD Biosciences, catalog no.749283), CD169 (BV421, 3D6.112 clone; BioLegend, catalog no. 142421), and major histocompatibility complex class II (PE-Cy5, M5/114.15.2 clone; BioLegend, catalog no. 107611). Samples were analyzed by flow cytometry on a BD Symphony A3 and analyzed on FlowJo.

### Whole-mouse imaging of vaccination drainage

Mice were immunized subcutaneously at the tail base with fluorescently labeled antigen or adjuvant. Immunizations were prepared as described, using fluorescently labeled components as indicated. For studies including fluorescently labeled components, immunizations were prepared by loading antigen onto alum in sterile TBS (Sigma-Aldrich, catalog no. T5912) for 30 min on a tube rotator before adding coadjuvants and incubating for 30 min on a tube rotator. Alum was labeled using 0.1 nmol of pSer_4_–Alexa Fluor 488. Imaging was completed using a PerkinElmer Xenogen Spectrum in vivo imaging system (IVIS), and the fluorescent signal at the injection site was quantified using Living Image software. The radiant efficiency was tracked longitudinally to monitor drainage from the injection site.

### Microscopy

Alum was incubated with Alexa Fluor 555–labeled pSer_4_-RBDJ or pSer_4_–Alexa Fluor 488 at 25°C for 30 min in TBS. These solutions were mixed and incubated together for 2 days before imaging. Fluorescence images were acquired on an Applied Precision DeltaVision microscope with a 100×/1.4 oil objective using the accompanying Softworx software. Image analysis was performed using Fiji (ImageJ version 2.1.0) by converting the images into a binary image, applying a Watershed transform, counting the number of particles (3D Objects Counter), and applying the colocalization threshold analysis to assess the number of particles for which there is colocalization of the two fluorescent signals. The number of alum particles with fluorescent colocalization was divided by the total number of alum particles detected in the image and reported as the fraction of particles with fluorescent colocalization.

### Statistical analysis

All data were plotted and all statistical analyses were performed using GraphPad Prism 8 software (La Jolla, CA). All graphs display mean values, and the error bars represent the SD unless otherwise specified. No samples or animals were excluded from the analyses. Statistical comparison was performed using a one-way analysis of variance (ANOVA) followed by Tukey’s post hoc test for single–time point data and two-way ANOVA followed by Tukey’s post hoc test for multi–time point longitudinal data. Statistical analysis of antibody titer was completed using log-transformed data. Data were considered statistically significant if the *P* value was less than 0.05.

## Notes

View/request a protocol for this paper from *Bio-protocol*.
