# Supplementary material for: Phosphate-mediated coanchoring of RBD immunogens and molecular adjuvants to alum potentiates humoral immunity against SARS-CoV-2
Source: Sci Adv. 2021 Dec 8;7(50):eabj6538. doi: 10.1126/sciadv.abj6538 (PMC8654298; doi:10.1126/sciadv.abj6538)
Supplement: Supplementary file 1 — Table S1 Figs. S1 to S10 [file sciadv.abj6538_sm.pdf]

Science Advances
AAAS

Supplementary Materials for

Phosphate-mediated coanchoring of RBD immunogens and molecular adjuvants to alum potentiates humoral immunity against SARS-CoV-2

Kristen A. Rodrigues, Sergio A. Rodriguez-Aponte, Neil C. Dalvie, Jeong Hyun Lee, Wuhbet Abraham, Diane G. Carnathan, Luis E. Jimenez, Julia T. Ngo, Jason Y. H. Chang, Zeli Zhang, Jingyou Yu, Aiquan Chang, Catherine Nakao, Benjamin Goodwin, Christopher A. Naranjo, Libin Zhang, Murillo Silva, Dan H. Barouch, Guido Silvestri, Shane Crotty, J. Christopher Love, Darrell J. Irvine*

*Corresponding author. Email: djirvine@mit.edu

Published 8 December 2021, *Sci. Adv.* **7**, eabj6538 (2021)
DOI: 10.1126/sciadv.abj6538

This PDF file includes:

Table S1
Figs. S1 to S10

Protein	Sequence
RBD<br>C-terminal cysteine	ITNLCPFGEVFNATRFASVYAWNRKRISNCVADYS<br>VLYNSASFSTFKCYGVSPTKLNDLCFTNVYADSFV<br>IRGDEVRQIAPGQTGKIADYNYKLPDDFTGCVIAW<br>NSNNLDSKVGGNYNYLYRLFRKSNLKPFERDISTE<br>IYQAGSTPCNGVEGFNCYFPLQSYGFQPTNGVGYQ<br>PYRVVVLSFELLHAPATVCGPKKSTNHHHHHHC
RBD<br>N-terminal cysteine	CITNLCPFGEVFNATRFASVYAWNRKRISNCVADY<br>SVLYNSASFSTFKCYGVSPTKLNDLCFTNVYADSF<br>VIRGDEVRQIAPGQTGKIADYNYKLPDDFTGCVIA<br>WNSNNLDSKVGGNYNYLYRLFRKSNLKPFERDIST<br>EIYQAGSTPCNGVEGFNCYFPLQSYGFQPTNGVGY<br>QPYRVVVLSFELLHAPATVCGPKKSTNHHHHHH
RBDJ<br>N-terminal cysteine	CITNLCPFGEVFNATRFASVYAWNRKRISNCVADY<br>SVLYNSASFSTFKCYGVSPTKLNDLCFTNVYADSF<br>VIRGDEVRQIAPGQTGKIADYNYKLPDDFTGCVIA<br>WNSNNLDSKVGGNYNYKYRLFRKSNLKPFERDIST<br>EIYQAGSTPCNGVEGFNCYWPLQSYGFQPTNGVGY<br>QPYRVVVLSFELLHAPATVCGPKKSTN
RBDJ-Blue<br>N-terminal cysteine	CITNLCPFGEVFNATRFASVYAWNRKRISNCVADY<br>SVLYNSASFSTFKCYGVSPTKLNDLCFTNVYADSF<br>VIRGDEVRQIAPGQTGKIADYNYKLPDDFTGCVIA<br>WNSNNLDSKVGGNYNYKYRLFRKSNLKPFERDIST<br>EIYQAGSTPCNGVEGFNCYWPLQSYGFQPTNGVGY<br>QPYRVVVLSFELLHAPATVCGPKKSTNDGGDGGGS<br>FIEDLLFNKVTLAD
RBDJ-PADRE<br>N-terminal cysteine	CITNLCPFGEVFNATRFASVYAWNRKRISNCVADY<br>SVLYNSASFSTFKCYGVSPTKLNDLCFTNVYADSF<br>VIRGDEVRQIAPGQTGKIADYNYKLPDDFTGCVIA<br>WNSNNLDSKVGGNYNYKYRLFRKSNLKPFERDIST<br>EIYQAGSTPCNGVEGFNCYWPLQSYGFQPTNGVGY<br>QPYRVVVLSFELLHAPATVCGPKKSTNDGGDGGGA<br>KFVAAWTLKAA

Table S1: Antigen sequences.

The amino acid sequences of RBD antigens used in these studies.

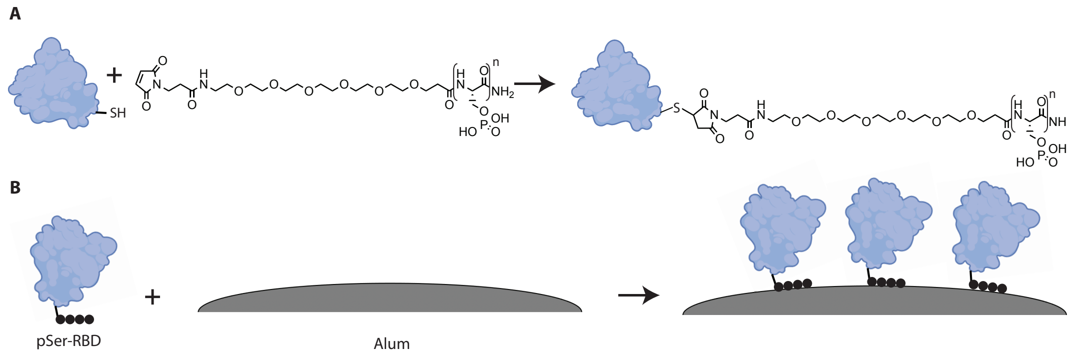

Fig. S1. pSer-modification of RBD antigens facilitates anchoring to alum.
(**A**) RBD antigens were expressed with terminal cysteines which can be coupled to short peptide linkers consisting of an N-terminal maleimide group and C-terminal pSer residues separated by a 6-unit poly(ethylene glycol) spacer. (**B**) pSer-modified RBD antigens are anchored to alum via ligand exchange between the phosphates in the pSer residues and hydroxyls on the surface of alum.

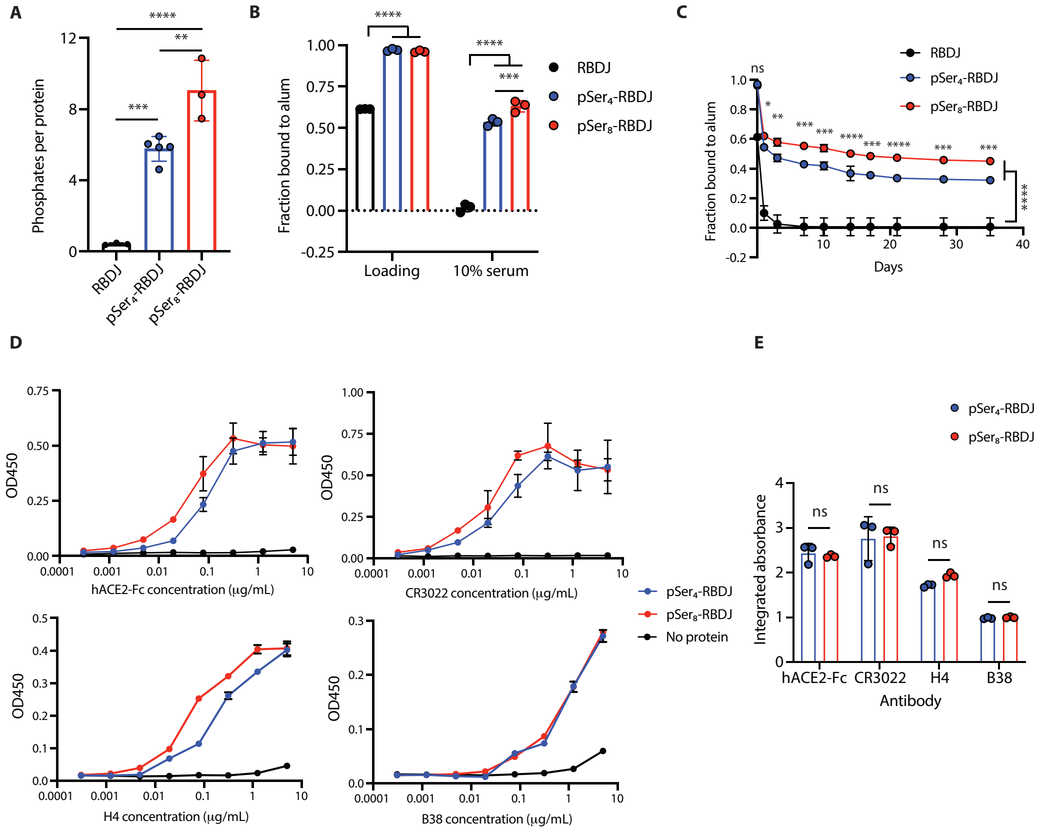

Fig. S2. pSer valency enables tuning of antigen-alum binding and influences humoral immune responses.

(**A**) RBDJ antigens with pSer$_4$ or pSer$_8$ peptides conjugated at the N-terminus were assayed for phosphates per protein by a malachite green assay. Statistical significance was determined by one-way ANOVA followed by Tukey's post-hoc test. (**B**) Unmodified, pSer$_4$-, or pSer$_8$-conjugated RBDJ were mixed with alum, and the fraction of protein bound to alum was assessed before ("Loading") and after incubation for 24 hours in 10% mouse serum at 37°C. Statistical significance was determined by one-way ANOVA followed by Tukey's post-hoc test. (**C**) Unmodified, pSer$_4$-, or pSer$_8$-conjugated RBDJ were mixed with alum and incubated in 10% mouse serum at 37°C. The fraction of protein bound to alum was assessed longitudinally. Statistical significance was determined by two-way ANOVA followed by Tukey's post-hoc test. (**D**) A modified sandwich ELISA approach was used to analyze the antigenicity profile of pSer-modified RBDJ. Shown are binding profiles of hACE2-Fc (top left), CR3022 (top right), H4 (bottom left), and B38 (bottom right) to RBDs captured on alum-coated plates (*n*=3 replicates), and the area under individual binding curves (**E**). Statistical significance was determined by two-way ANOVA followed by Sidak's multiple comparison test. Values plotted are means ± standard deviation. ns p>0.05, * p<0.05, ** p<0.01, *** p<0.001, **** p<0.0001.

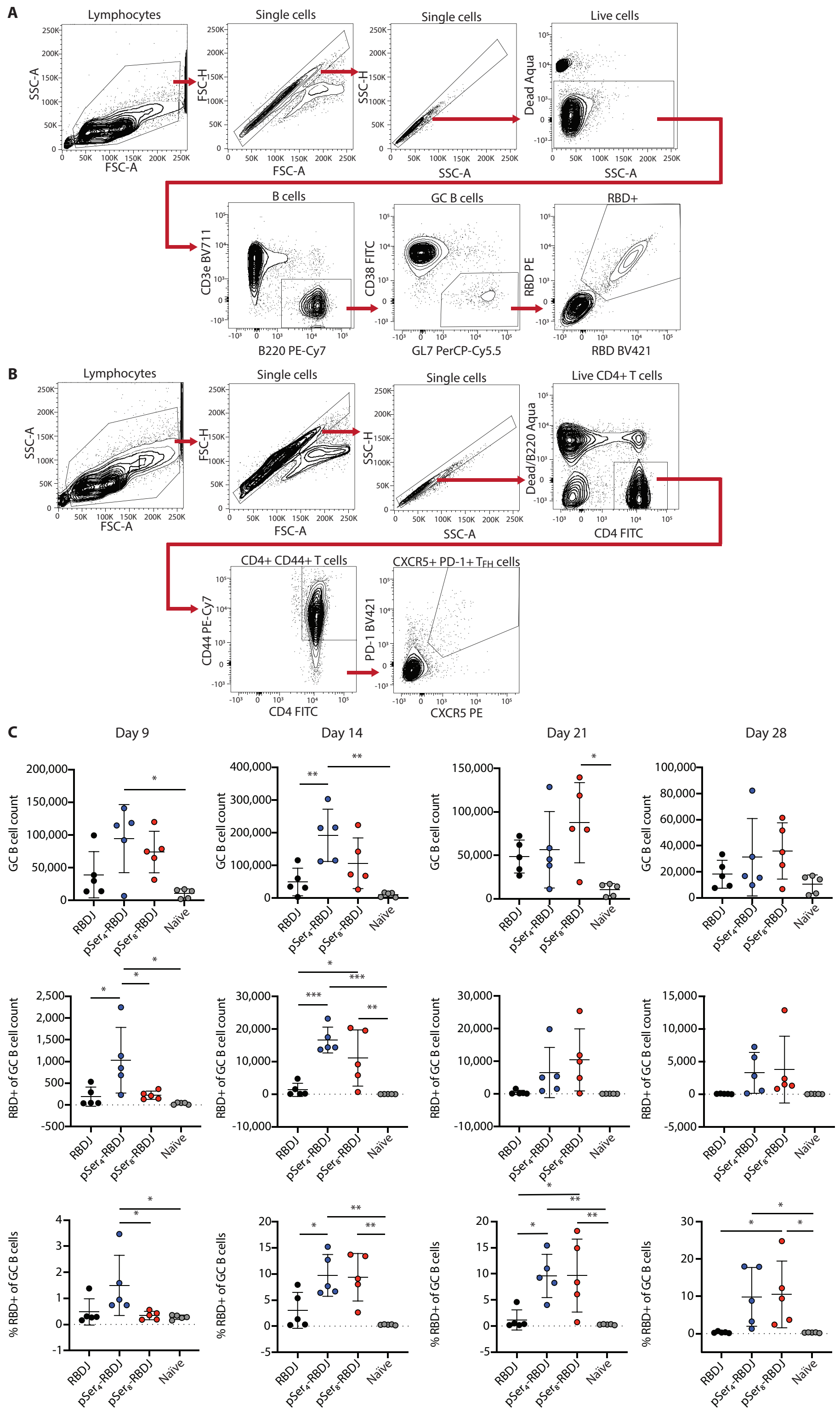

Fig. S3. pSer valency influences germinal center responses.

Representative flow cytometry gating plots of (**A**) RBD-specific germinal center (GC) B cell, and (**B**) T follicular helper (T_{FH}) cell staining. (**C**) BALB/c mice (*n*=5 animals/group) were

immunized with 10 μg unmodified, $pSer_4$-, or $pSer_8$-conjugated RBDJ and 100 μg alum, and germinal center (GC) responses in draining inguinal lymph nodes were analyzed by flow cytometry over time for total GC B cell counts (top), RBD-specific GC B cell counts (middle), and percent RBD-specific GC B cells (bottom) at 9, 14, 21, and 28 days post-immunization. Statistical significance was determined by two-way ANOVA followed by Tukey's post-hoc test. Values plotted are means ± standard deviation. ns $p>0.05$, * $p<0.05$, ** $p<0.01$, *** $p<0.001$, **** $p<0.0001$.

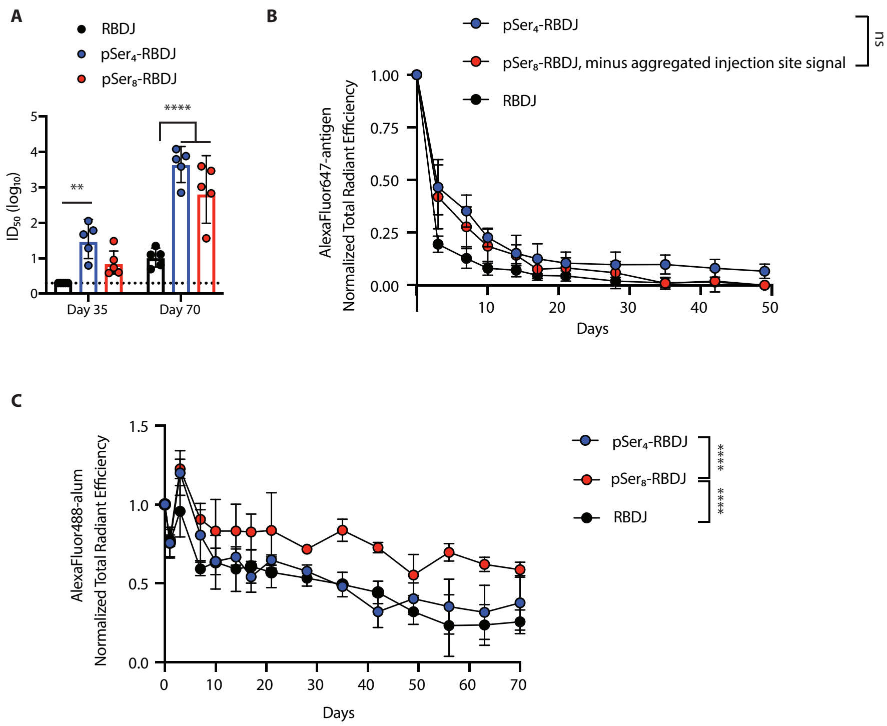

Fig. S4. pSer-RBDJ drainage is a combination of antigen-alum complex trafficking and release of antigen from alum at the injection site.

(**A**) BALB/c mice (*n*=5 animals/group) were immunized with 10 µg unmodified, pSer4-, or pSer8-conjugated RBDJ and 100 µg alum and boosted at 6 weeks. Half-maximal inhibitory titers (ID_{50}) values were assessed for hACE2-RBD interactions at day 35 and day 70. The dashed line indicates the limit of detection. Values plotted are geometric means ± geometric standard deviation. Statistical significance was determined by two-way ANOVA followed by Sidak's multiple comparisons test. (**B**) Mice were immunized with 10 µg fluorescently labeled RBDJ plus alum and the fluorescence at the injection site was quantified longitudinally (*n*=4 animals/group), as in **Fig. 3A-B**. The signal remaining at the injection site at day 49 for pSer8-RBDJ was subtracted from the longitudinal pSer8-RBDJ signal and plotted for comparison to pSer4-RBDJ. Values plotted are means ± standard deviation. Statistical significance between pSer4-RBDJ and pSer8-RBDJ was determined by two-way ANOVA followed by Tukey's post-hoc test. (**C**) Mice were immunized with 10 µg RBDJ plus 100 µg of labeled alum and the fluorescence at the injection site was quantified longitudinally (*n*=3 animals/group). Values plotted are means ± standard deviation. Statistical significance was determined by two-way ANOVA followed by Tukey's post-hoc test. ns $p>0.05$, * $p<0.05$, ** $p<0.01$, *** $p<0.001$, **** $p<0.0001$.

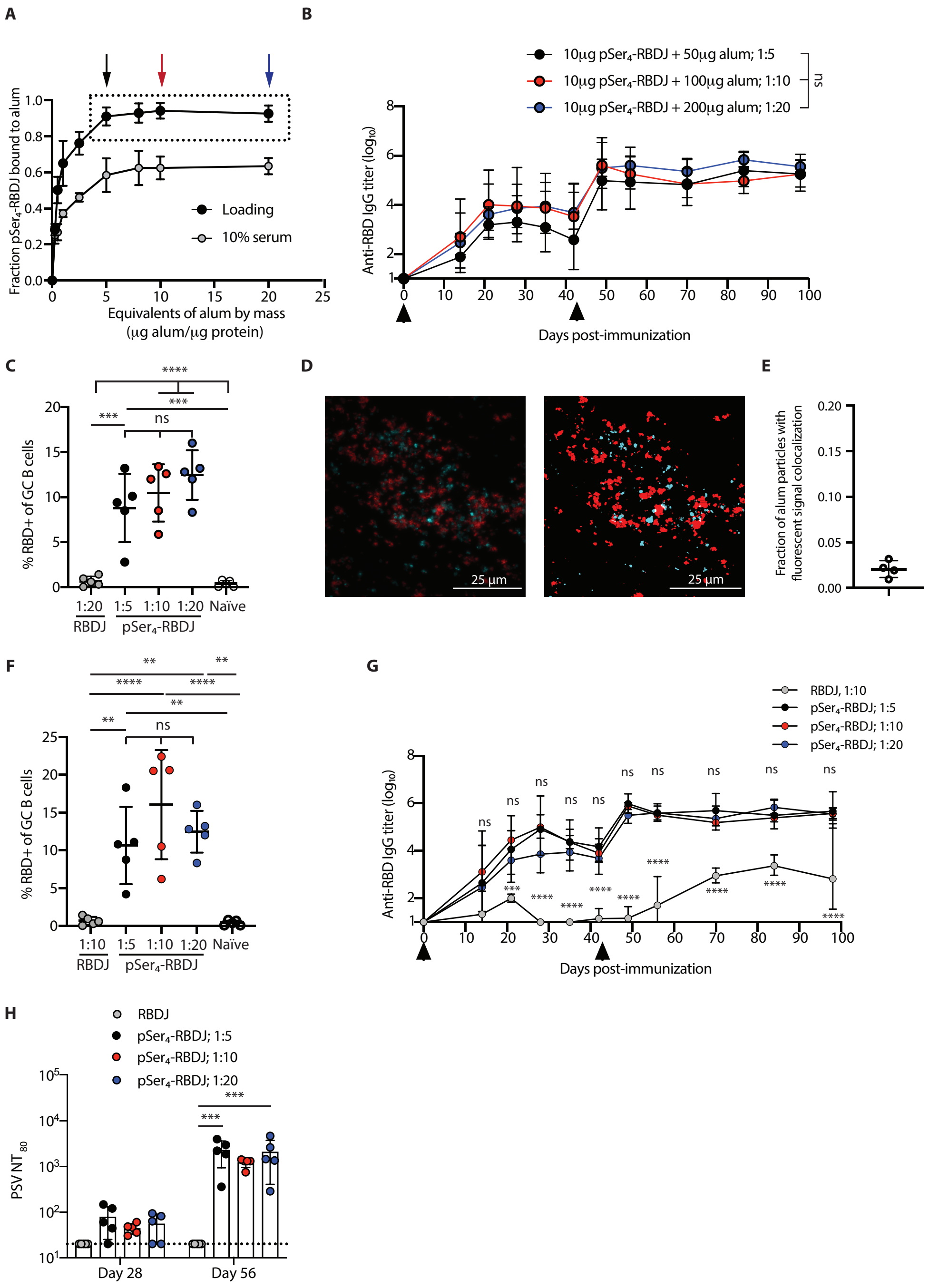

Fig. S5. Average antigen density of pSer-RBDJ on alum does not significantly alter humoral responses.

(**A**) $pSer_4$-RBDJ was mixed with alum in varying ratios, and the fraction of protein bound to alum was assessed before ("Loading") and after incubation for 24 hours in 10% mouse serum at 37°C. Values plotted are means ± standard deviation. Arrows indicate ratios selected for further evaluation. (**B**) BALB/c mice (*n*=5 animals/group) were immunized with 10 µg $pSer_4$-RBDJ and varying amounts of alum and boosted at 6 weeks. Serum IgG antibody responses were assessed longitudinally by ELISA. Arrows indicate immunization time points. Values plotted are

geometric means ± geometric standard deviation. Statistical significance was determined by two-way ANOVA followed by Tukey's post-hoc test. (**C**) Mice (*n*=5 animals/group) were immunized with 10 μg unmodified or $pSer_4$-conjugated RBDJ and varying amounts of alum, and germinal center (GC) responses in draining inguinal lymph nodes were analyzed by flow cytometry at day 14 post-immunization. Values plotted are means ± standard deviation. Statistical significance was determined by two-way ANOVA followed by Tukey's post-hoc test. (**D**) Alum loaded with AlexaFluor647-labeled $pSer_4$-RBDJ were mixed with alum labeled with $pSer_4$-AlexaFluor488 at low density and incubated together for 2 days prior to imaging (left). Representative image shown. The fluorescence overlap was assessed (right) and (**E**) the fraction of alum particles with fluorescent signal colocalization was measured. Values plotted are means ± standard deviation. (**F**) Mice (*n*=5 animals/group) were immunized with 10 μg $pSer_4$-RBDJ plus 200 μg alum at varying average antigen densities. Immunizations were prepped by first loading $pSer_4$-RBDJ on alum at the indicated ratios and then supplementing alum just prior to immunization such that all groups received an equal alum dose. GC responses in draining inguinal lymph nodes were analyzed by flow cytometry at day 14 post-immunization. Values plotted are means ± standard deviation. Statistical significance was determined by two-way ANOVA followed by Tukey's post-hoc test. (**G**) Mice (*n*=5 animals/group) were immunized with 10 μg $pSer_4$-RBDJ plus 200 μg alum at varying average antigen densities. Serum IgG antibody responses were assessed longitudinally by ELISA. Arrows indicate immunization time points. Values plotted are geometric means ± geometric standard deviation. Statistical significance was determined by two-way ANOVA followed by Tukey's post-hoc test. Statistical comparisons between all $pSer_4$-RBDJ groups are denoted above plot, while statistical comparison between each $pSer_4$-RBDJ group against the RBDJ group is denoted between $pSer_4$-RBDJ groups and RBDJ group. (**H**) Serum SARS-CoV-2 pseudovirus neutralizing titer ID_{80} (PSV NT_{80}) were assessed for serum collected at day 28 and day 56. The dashed line indicates the limit of detection. Values plotted are means ± standard deviation. Statistical significance was determined by two-way ANOVA followed by Tukey's post-hoc test. ns $p>0.05$, * $p<0.05$, ** $p<0.01$, *** $p<0.001$, **** $p<0.0001$.

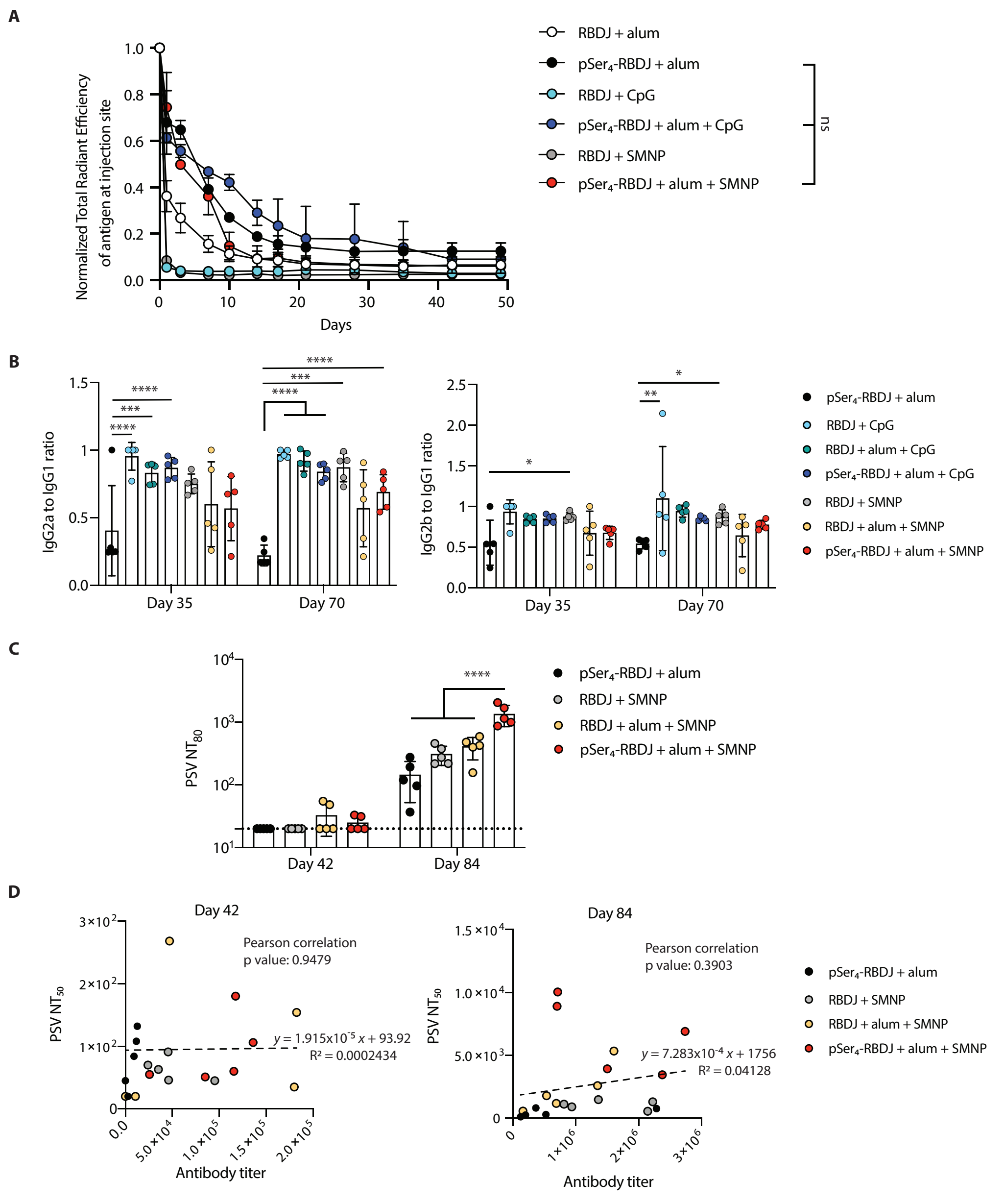

Fig. S6. Co-adjuvants SMNP and CpG promote balanced antibody isotype responses and enhance humoral responses.

(**A**) BALB/c mice were immunized with 10 µg fluorescently labeled RBDJ with or without alum plus co-adjuvants CpG or SMNP, and the fluorescence at the injection site was quantified longitudinally (*n*=3 animals/group). Values plotted are means ± standard deviation. Statistical significance between pSer$_4$-RBDJ groups was determined by one-way ANOVA followed by Tukey's post-hoc test. (**B**) Mice (*n*=5 animals/group) were immunized with 10 µg unmodified RBDJ or pSer$_4$-RBDJ and 100 µg alum and/or 30 µg CpG or 5 µg SMNP and boosted at 6 weeks, as in **Fig 4E-G**. The ratio of IgG2a to IgG1 (left) and IgG2b to IgG1 (right) were calculated at day 35 and day 70. Values plotted are means ± standard deviation. Statistical significance was determined by two-way ANOVA followed by Tukey's post-hoc test. (**C**) Mice (*n*=5 animals/group) were immunized with 10 µg unmodified RBDJ or pSer$_4$-RBDJ and 100 µg

alum and/or 5 μg SMNP and boosted at 6 weeks, as in **Fig 4G**. Serum SARS-CoV-2 pseudovirus neutralizing titer ID_{80} (PSV NT_{80}) were assessed for serum collected at day 42 and day 84. The dashed line indicates the limit of detection. Values plotted are means ± standard deviation. Statistical significance was determined by two-way ANOVA followed by Tukey's post-hoc test. (**D**) Mice (*n*=5 animals/group) were immunized with 10 μg unmodified RBDJ or $pSer_4$-RBDJ and 100 μg alum and/or 5 μg SMNP and boosted at 6 weeks, as in **Fig 4G**. Plotted is the binding titer versus the neutralizing titer for both day 42 and 84. The corresponding linear fit is plotted as a dashed line, and the Pearson correlation was assessed for each timepoint. ns $p>0.05$, * $p<0.05$, ** $p<0.01$, *** $p<0.001$, **** $p<0.0001$.

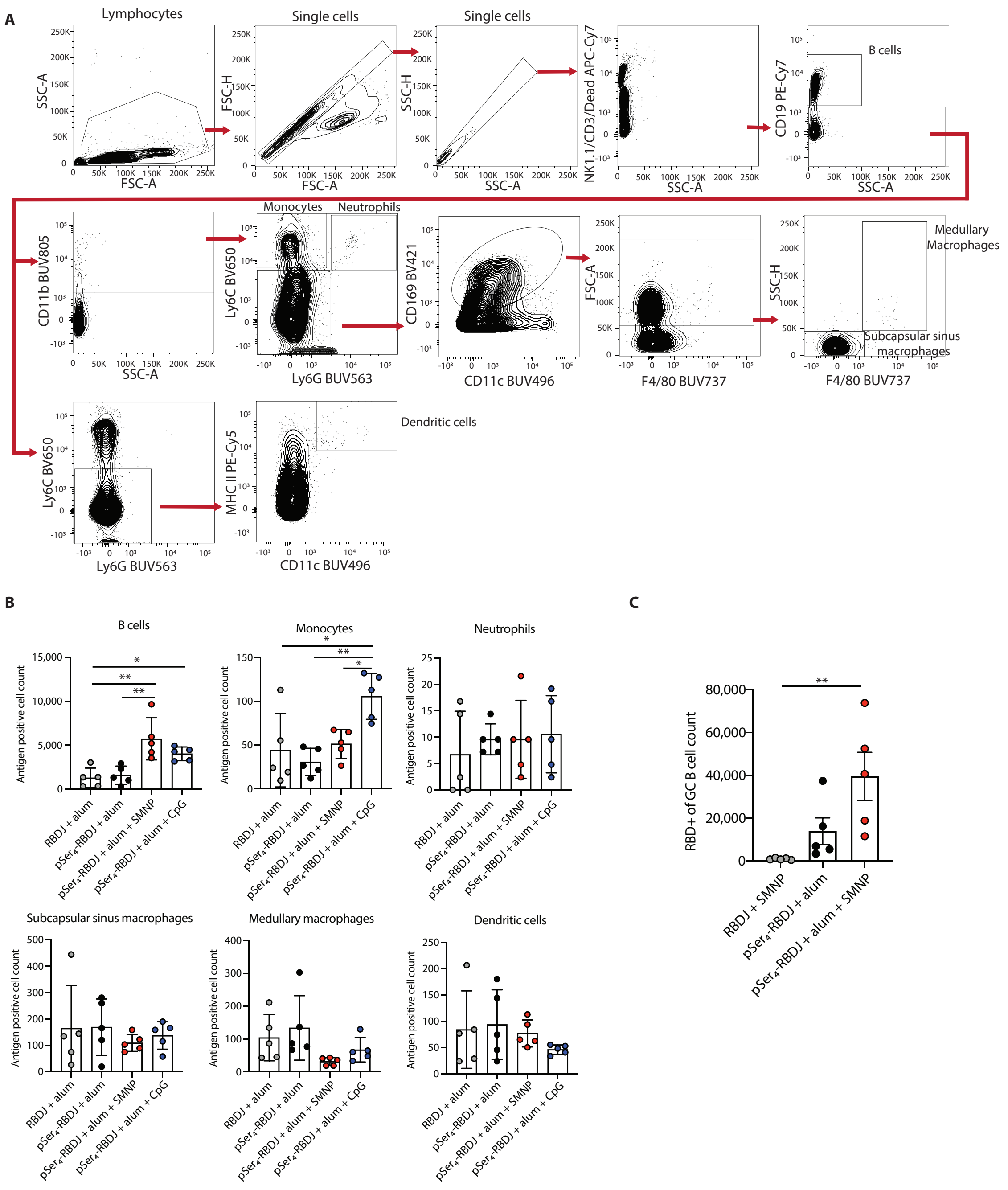

Fig. S7. Co-adjuvants enhance antigen uptake and germinal center responses

BALB/c mice (*n*=5 animals/group) were immunized with 10 µg AlexaFluor555 labeled antigen and 100 µg alum and 5 µg SMNP or 30 µg CpG, and the inguinal lymph nodes were collected 7 days post-immunization. (**A**) Representative flow cytometry gating plots. (**B**) The number of cells positive for AlexaFluor555 labeled antigen is plotted for B cells, monocytes, neutrophils, subcapsular sinus macrophages, medullary macrophages, and dendritic cells. Values plotted are means ± standard deviation. Statistical significance was determined by one-way ANOVA followed by Tukey's post-hoc test. (**C**) Mice (*n*=5 animals/group) were immunized with 10 µg unmodified RBDJ or $pSer_4$-RBDJ and 100 µg alum and/or 5 µg SMNP and the RBD-specific

germinal center (GC) B cell responses in the draining inguinal lymph nodes were analyzed by flow cytometry at day 14 post-immunization. Values plotted are means ± standard error of the mean. Statistical significance was determined by one-way ANOVA followed by Tukey's post-hoc test. ns $p>0.05$, * $p<0.05$, ** $p<0.01$, *** $p<0.001$, **** $p<0.0001$.

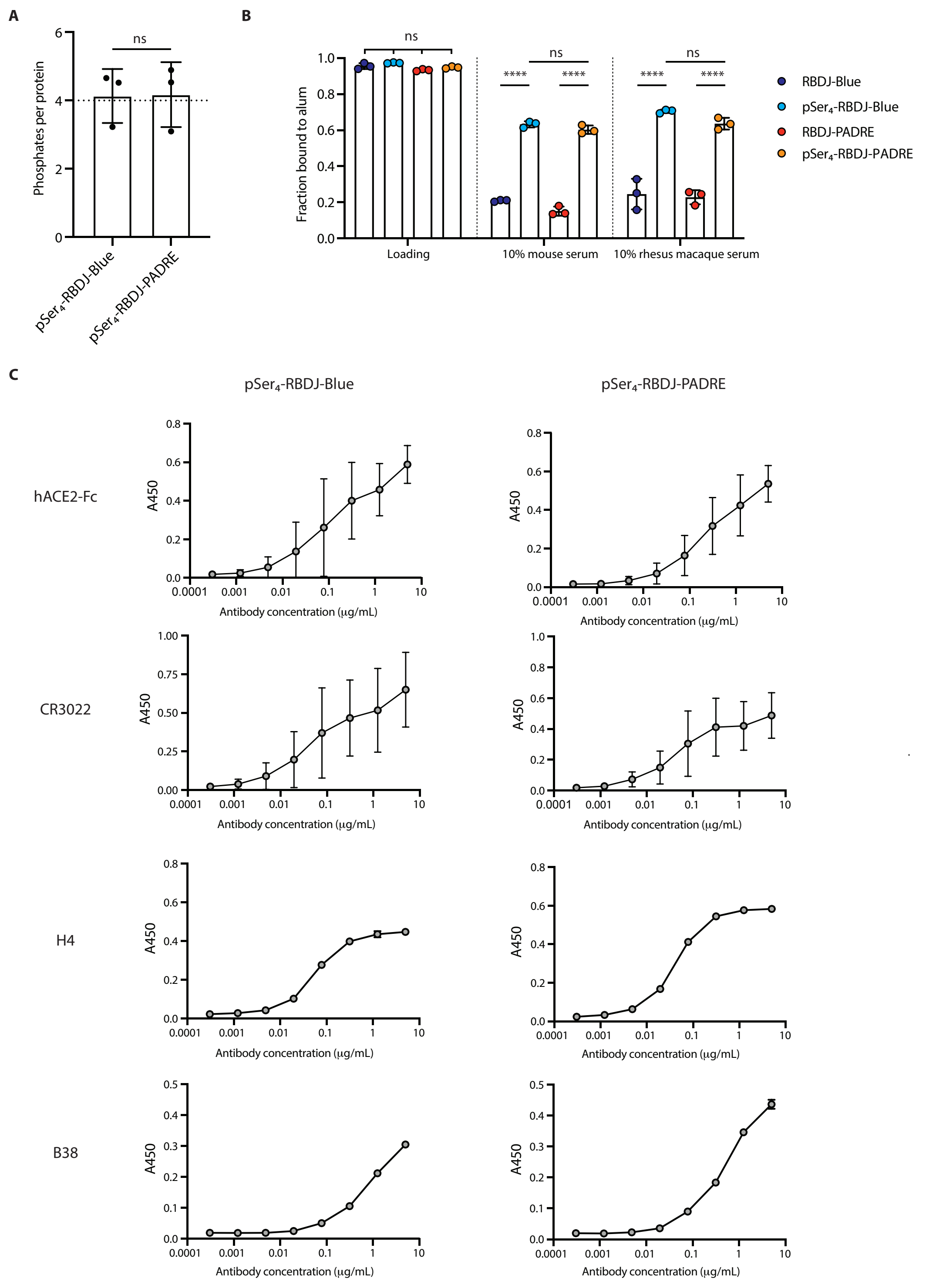

Fig. S8. pSer-RBDJ constructs with added T helper epitopes exhibit strong binding and retained antigenicity on alum.

(**A**) pSer4-conjugated RBDJ antigens with added Blue and PADRE T helper epitopes were assayed for phosphates per protein by a malachite green assay. Statistical significance was determined by Mann-Whitney test. (**B**) pSer4-conjugated or unmodified RBDJ antigens with

added Blue and PADRE T helper epitopes were mixed with alum, and the fraction of protein bound to alum was assessed before (“Loading”) and after incubation for 24 hours in 10% mouse or rhesus macaque serum at 37°C. Statistical significance was determined by one-way ANOVA followed by Tukey’s post-hoc test. (**C**) A modified sandwich ELISA approach was used to analyze the antigenicity profile of $pSer_4$-conjugated RBDJ antigens with added Blue and PADRE T helper epitopes. Shown are binding profiles of hACE2-Fc (first row), CR3022 (second row), H4 (third row), and B38 (bottom row) to $pSer_4$-RBDJ-Blue (left) and $pSer_4$-RBDJ-PADRE (right) captured on alum-coated plates (n=3 replicates). Values plotted are means ± standard deviation. ns $p>0.05$, **** $p<0.0001$.

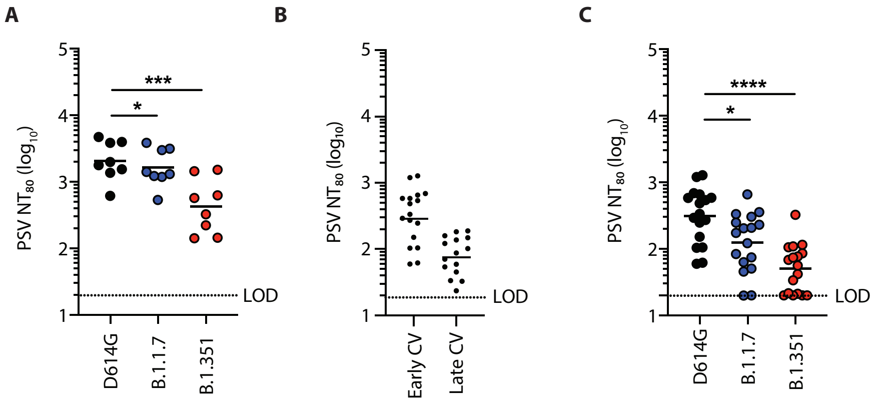

Fig. S9. pSer-RBDJ:alum:SMNP immunization generates robust neutralizing responses against circulating SARS-CoV-2 variants in rhesus macaques.

Rhesus macaques were immunized with 50 µg pSer-RBDJ-Blue (*n*=4 animals) or 50 µg pSer-RBDJ-PADRE (*n*=4 animals) combined with 0.5 mg alum and 50 µg SMNP s.c. in the left and right deltoid at week 0 and week 6. Sera were collected over the course of the immune response. (**A**) Serum SARS-CoV-2 pseudovirus neutralizing titer ID_{80} (PSV NT_{80}) at week 8 post-immunization to SARS-CoV-2 D614G, B.1.1.7, and B.1.351 pseudoviruses. Geometric mean titer is shown by the black line. (**B**) Neutralization titers of convalescent (CV) human serum samples are shown for comparison. Early CV samples are from an average of 32.6 days post symptom onset (range 21-43 days, median 33 days), and late CV samples are from an average of 178.7 days post symptom onset (range 151-221 days, median 179). (**C**) Early CV serum PSV NT_{80} titers to D614G, B.1.1.7, and B.1.351 pseudoviruses. Friedman test followed by Dunn's multiple comparisons test was conducted to compare serum binding or neutralization titers to B.1.1.7 and B.1.351 isolates relative to the D614G control. Limit of detection (LOD) indicates the lowest serum dilution tested. ns $p>0.05$, * $p<0.05$, ** $p<0.01$, *** $p<0.001$, **** $p<0.0001$.

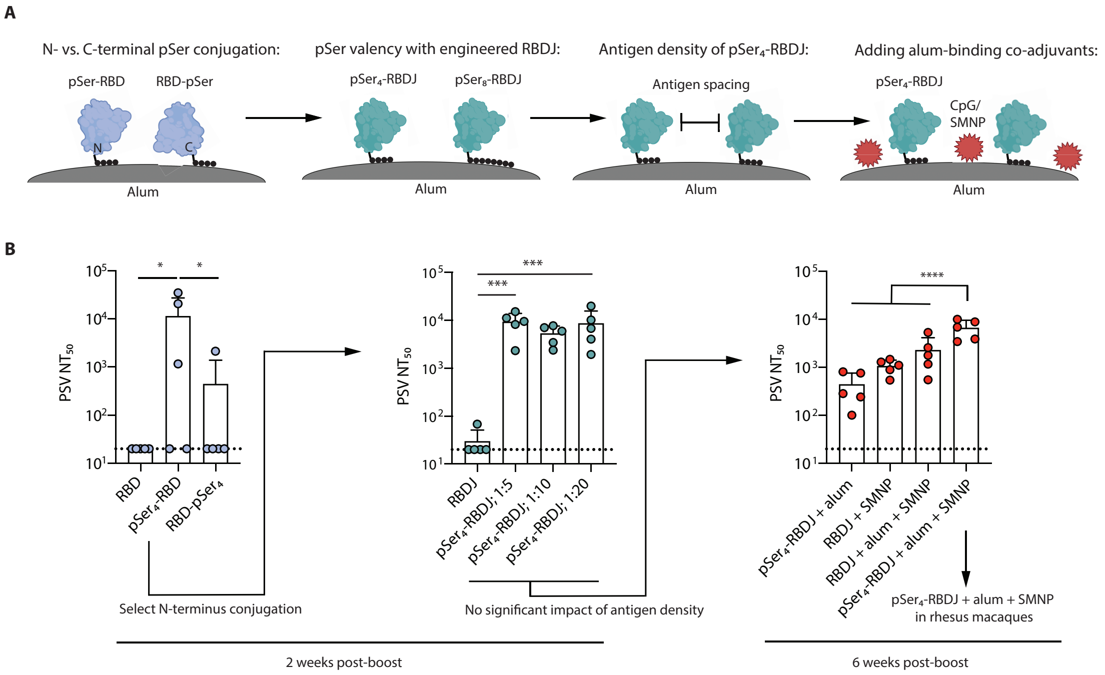

Fig. S10. Overview of immunization platform and optimization strategy.

(**A**) Outline of the iterations of the immunization platform. From left to right, we first investigated the impact of N- versus C-terminus pSer conjugation to RBD, moving forward with the N-terminal pSer conjugation approach. Next, we assessed the role of pSer valency with an engineered RBD protein called RBDJ and tested the impact of antigen density on alum. Finally, we added alum-binding co-adjuvants to investigate synergistic enhancement of responses. (**B**) The corresponding post-boost serum SARS-CoV-2 pseudovirus neutralizing titer ID_{50} (PSV NT_{50}) for relevant groups, reproduced from **Fig. 2C**, **fig. S5H**, and **Fig. 4J.** The dashed line indicates the limit of detection. Values plotted are means ± standard deviation. Statistical significance was determined by two-way ANOVA followed by Tukey's post-hoc test. ns $p>0.05$, * $p<0.05$, ** $p<0.01$, *** $p<0.001$, **** $p<0.0001$.
